# Morphological features and molecular mechanisms in peritoneal adhesions from patients with chronic abdominal postoperative pain

**DOI:** 10.1016/j.ebiom.2025.105746

**Published:** 2025-05-23

**Authors:** Masja Karina Toneman, Priscilla Pauline Marianne Faas, Maurits Johan Christian Antoine Marie Gielen, Valentina Carotti, Anne Elisa Adriana van Oirschot, Judith Petronella Maria Mangnus, Anne Wintjens, Casper Mihl, Josephine Huige, Bianca Grosser, Marjolein Blussé van Oud-Alblas, Koen Willem van Dongen, Nicole Dorine Bouvy, Harry van Goor, Daniel Keszthelyi, Heike Irmgard Grabsch, Richard Peter Gerardus ten Broek

**Affiliations:** a Surgery Department, Radboudumc, Nijmegen, the Netherlands; b Surgery Department, Maastricht University Medical Centre+, Maastricht, the Netherlands; c GROW-School for Oncology and Reproduction, Maastricht University, 6229 ER, Maastricht, the Netherlands; d Physiology Department, Radboudumc, Nijmegen, the Netherlands; e Radiology Department, Maastricht University Medical Centre+, Maastricht, the Netherlands; f CARIM School for Cardiovascular Diseases, Maastricht University, Universiteitssingel 50, PO Box 616, 6200 MD, Maastricht, the Netherlands; g Radiology Department, Rijnstate Hospital, Arnhem, the Netherlands; h Pathology Department, Medical Faculty Augsburg, University of Augsburg, Augsburg, Germany; i Surgery Department, Maasziekenhuis Pantein, Boxmeer, the Netherlands; j Gastroenterology-hepatology Department, Maastricht University Medical Centre+, Maastricht, the Netherlands; k Division of Pathology and Data Analytics, Leeds Institute of Medical Research at St James's University, University of Leeds, Leeds, UK; l Surgery Department, Leiden University Medical Centre, Leiden, the Netherlands

**Keywords:** General surgery, Abdominal pain, Histology, Immunohistochemistry, Gene expression

## Abstract

**Background:**

Chronic abdominal pain affects 10–20% of all patients following abdominal surgery, with adhesions as a predominant cause. However, the biological mechanisms underlying adhesion-related pain are not fully elucidated. This study aimed to establish the morphological and molecular phenotype of adhesions in patients with and without chronic postoperative abdominal pain.

**Methods:**

In this case–control study, biopsies of adhesions were obtained from patients with chronic postoperative abdominal pain (related to adhesions on cineMRI) and controls without pain, from two tertiary care and one secondary care hospital. Quantitative histological analysis of haematoxylin and eosin-stained sections was performed, while immunohistochemical (IHC) markers for nerve tissue (S100, calretinin and synaptophysin) were quantified through image analysis. RNA expression of genes (*TRPV1*, *BDNF*, *TAC1*, *TACR1*, *NGF*) was measured using real time quantitative polymerase chain reaction (RT-qPCR). Controls were matched to cases by sex, age, and prior surgery, accepting small variations due to patient availability. An independent two-sided t-test was used to detect differences in IHC and RT-qPCR analysis between groups.

**Findings:**

Adhesions from 31 patients with pain were compared to those from 31 patients without pain, consisting of 48% connective tissue and 41% adipose tissue. Immunohistochemical analysis revealed increased nerve tissue in patients with pain (S100: median 597 ppm (range 92.2–3223.2 ppm) vs 151 ppm (range 15.2–1683.8 ppm) p < 0.001; calretinin: median 463 ppm (range 72.7–2996.5 ppm) vs 275 ppm (range 35.3–3194.8 ppm) p = 0.040). *NGF* showed a higher mRNA expression in adhesions from patients with pain compared to controls (p = 0.012).

**Interpretation:**

This study suggests a distinct morphological and molecular phenotype of adhesions in patients experiencing adhesion-related pain, providing insights into underlying mechanisms.

**Funding:**

Veni grant from The Dutch Governmental Organisation for Health Research and Development (ZonMw grant number 91619035).

**Research in context**

**Evidence before this study**

Chronic abdominal pain following surgery leads to a significant reduction in quality of life. Adhesions, which form as fibrous connections between abdominal organs and the abdominal wall, are the most common pathology linked to chronic post-operative pain. However, the direct role of adhesions in pain development remains controversial. Previous studies suggest that adhesions may cause pain through indirect mechanisms, such as restricting organ mobility and stimulating visceral stretch receptors. Others propose that adhesions may transmit pain stimuli directly via nerve fibres found within their structure.

In April 2025 we performed a scoping review in PubMed, EMBASE and Web of Science for papers exploring mechanisms of pain development in patients with adhesions including the mesh terms (“Tissue adhesions [mesh]” OR “intestinal disease/surgery [mesh]” OR “abdomen/surgery [mesh]” OR “peritoneum/surgery [mesh]) AND “abdominal pain [mesh]” OR “pelvic pain [mesh]”. To date, some studies found that adhesions can contain nerve fibres. However, no quantitative comparison of the amount of nerve tissue was made between adhesions from patients with or without chronic pain. Molecular studies of adhesions have focused on the acute formation of adhesions, leaving the mechanisms of chronic pain development poorly understood.

**Added value of this study**

This study provides a comprehensive quantitative comparison of nerve density and molecular expression between adhesions in patients with chronic abdominal pain and those without pain. Patients with chronic pain experienced continuous or intermittent pain daily, causing disability or requiring analgesics, and cineMRI was used to correlate symptoms with adhesions. Controls had a matching history of abdominal surgery and were suspected to have adhesions, but were scheduled for non-pain-related surgeries. Controls were excluded if they had chronic pain or lacked adhesions intraoperatively.

We found a significantly higher nerve density in adhesions from patients with chronic pain, alongside elevated gene expression of *NGF*, a neurotrophic factor involved in both adhesion formation and pain signalling. These findings support the hypothesis that adhesions may contribute directly to chronic pain, possibly through neuropathic mechanisms. Additionally, the correlation between nerve density and the composition of adhesion tissue (connective tissue and blood vessels) suggests a unique morphological profile in adhesions associated with pain.

**Implications of all the available evidence**

This study contributes new insights into the pathophysiology of chronic abdominal pain related to adhesions. The identification of increased nerve density and *NGF* expression in pain-associated adhesions highlights potential targets for therapeutic intervention. Current treatment options, such as adhesiolysis with an adhesion barrier, are effective only for a subset of patients, while others rely on conservative pain management strategies. The findings of this study suggest that further research into neuropathic pain mechanisms could improve diagnosis and treatment for patients suffering from chronic postoperative pain. Moreover, understanding the molecular pathways involved in adhesion-related pain may open new potential for the development of targeted therapies that modulate neurotrophic factors.

## Introduction

In high-income countries, more than half of the population will undergo abdominal surgery for a variety of reasons during their lifetime.^1^^,^^2^ Following abdominal surgery, 10–20% of patients develop chronic pain, significantly impacting their long-term quality of life.^3^ Adhesions are the predominant pathology found in patients with chronic postoperative pain, yet causality remains a subject of ongoing debate.^4^, ^5^, ^6^ Adhesions are intra-abdominal fibrous connections between organs and the abdominal wall, forming as a result of scarring after tissue damage.

Previous studies showed the benefits of both adhesiolysis, which is the surgical dissection of adhesions, and the application of an adhesion barrier in selected patients with chronic abdominal pain related to adhesions.^7^^,^^8^ However, the mechanisms underlying adhesion-related pain are not yet fully understood and many patients with adhesions have no symptoms. A deeper comprehension of the biological mechanisms involved could improve the diagnosis and management of patients with chronic postoperative pain. This understanding may also facilitate the identification of patients suitable for adhesiolysis with adhesion barrier application, and the discovery of novel therapeutic targets for those currently relying on conservative treatment, which primarily relies on analgesia.^8^

Several hypotheses have been proposed to explain the relationship between chronic postoperative abdominal pain and adhesions.^9^, ^10^, ^11^, ^12^ One hypothesis suggests that adhesions induce pain indirectly by restricting organ mobility, possibly stimulating stretch receptors in the smooth muscle wall of intra-abdominal organs.^9^ This hypothesis is founded on the findings of an adhesive small bowel obstruction (ASBO). ASBO is an acute condition in which the bowel is *entangled in an adhesion resulting in severe acute pain, bowel distention and nausea.* Partial obstructions are hypothesised not to result in ASBO, but in distention and indirect pain. However, conscious laparoscopic pain mapping studies demonstrated that probing pelvic adhesions directly elicits pain responses in patients, suggesting that the adhesions themselves may be able to transmit pain stimuli.^10^^,^^11^^,^^13^ The presence of nerve fibres observed in histological studies of adhesions could contribute to the pain.^9^^,^^12^^,^^14^ However, previous studies have only qualitatively or semi-quantitatively evaluated nerve fibres in patients with adhesion-related pain.^9^^,^^12^^,^^14^ In recent years, there has been renewed scientific interest in the biology of adhesion formation. Although these studies have deepened our fundamental understanding of the complex cascade (involving multiple pathways) that results in adhesion formation, the biology of adhesion-related chronic pain remains to be elucidated.^15^^,^^16^ Chronic pain from adhesions might be associated with factors that relate to both wound healing and chronic pain such as nerve growth factor (NGF) and brain-derived neurotrophic factor (BDNF), both of which stimulate nerve growth.^17^^,^^18^ Nociceptive signalling appears to trigger the transcription and activation of transient receptor potential vanilloid 1 (TRPV1), which stimulates the release of substance P (SP), resulting in Neurokinin receptor 1 (NK1) signalling. These factors have previously been associated with the adhesion formation cascade.^19^^,^^20^ A sustained upregulation of such molecules as part of ongoing tissue remodelling could contribute to the direct transmission of pain stimuli by adhesions.

We hypothesised that patients with adhesion-related pain have more nerve fibres and a higher expression of nociception-associated genes in the adhesion tissue than those without pain. Therefore, the aim of this study was to assess morphological features and mRNA expression patterns of relevant candidate genes in adhesion tissue from patients with and without adhesion-related pain.

## Methods

### Patients

Patients from three hospitals were included between March 1st, 2019, and May 1st, 2022, in this prospective observational study. The study locations were Radboudumc, Nijmegen, the Netherlands; Maashospital Pantein, Boxmeer, the Netherlands; Maastricht University Medical Centre+, Maastricht, the Netherlands. These are specialised centres for chronic complaints after surgery and regularly receive referrals of patients with suspected adhesion-related symptoms from various facilities throughout the country. The expert centres closely collaborate and refer patients to each other for surgical treatment for logistic reasons, especially during the COVID period. In all patients, the most recent surgery occurred at least one year prior to inclusion in the study. The exclusion criteria were cancellation of surgery and perioperative absence of adhesions. Patients with chronic abdominal pain for at least 12 months, who were suspected of having adhesions and selected for operative treatment based on a predefined protocol, were eligible for inclusion in the pain group of this study.^8^ As part of this protocol a shared decision (surgeon and patient) to operate was made after carefully weighing the benefits and risks of surgery.^21^ Chronic postoperative abdominal pain was defined as daily pain complaints starting after abdominal surgery, causing pain-related disability and/or requiring the use of analgesics. Patients with intermittent episodes of pain related to ASBO were excluded. Symptoms were correlated to results of non-invasive cine magnetic resonance imaging (cineMRI), using a dedicated dynamic protocol aiming to visualise presence or absence of adhesions, and to establish the diagnosis of adhesion-related pain.^8^^,^^22^ A comprehensive description of cineMRI techniques can be found elsewhere.^23^ On cineMRI, visceral sliding is recorded using patient controlled breathing techniques. The absence of normal visceral sliding suggests presence of adhesions between abdominal organs and structures on that location. When the location of the adhesions on cineMRI clinically correlates with location of experienced abdominal pain, and other causes of pain have sufficiently been ruled out, the suspected diagnosis of adhesion-related chronic abdominal pain is established. For the control group, patients placed on the waiting list for elective surgery were screened on baseline variables for matching with pain patients. Patients were eligible to be included in the control group if there was a clinical suspicion of peritoneal adhesions based on their abdominal surgery history, and if they were scheduled for abdominal surgery for an indication unrelated to pain. The exclusion criteria for controls were any type of chronic pain or no adhesions found during surgery. Controls were 1:1 matched prospectively to the patients with pain, based on their sex, age, and number and category of previous surgical interventions. When a match on at least 80% of abovementioned baseline variables to a pain patient was identified, the patient was screened for eligibility as control, accepting small variations due to availability of eligible patients on the surgical schedule. For example, patient age at surgery was categorised (18–24, 25–44, 45–64, 65–84, ≥85), matched patients could be 52 and 61 but fall in the same age category.

### Ethics

The PAINPAD study was registered at ClinicalTrials.gov (NCT03938168) on 05-01-2019 and adhered to the Helsinki declaration. The study was ethically approved by the ethical committee of region Arnhem-Nijmegen (2018-4801) on November 14th, 2018. Written informed consent was obtained from all patients.

### Sample collection and storage

Tissue samples (biopsies) from adhesions were obtained during surgery for study purposes. A biopsy was obtained by securing the adhesion in place with a non-traumatic instrument and making incisions through the adhesions above and below the instrument. We aimed to take biopsies from the central portion of the adhesive tissue while avoiding the parietal or visceral peritoneum. In the case of localised pain complaints, adhesions were biopsied within a predetermined area of pain (target area) and, if available, at remote sites with adhesions (non-target area). For patients with diffuse pain (from the group of patients with chronic abdominal pain) or control patients (without pain), biopsies were taken from adhesions in a random area. Supplementary Figure S1 provides a visual representation of the adhesion biopsy approach and delineates abdominal areas for distinguishing locations of pain in target or non-target areas.

Per patient, at least one biopsy was stored in 4% paraformaldehyde (Added Pharma) in phosphate-buffered saline (pH 7.2) for processing in paraffin (Poth Hille) and subsequent histological assessment, and a different biopsy from the same adhesion was submerged into RNAlater® (Cat. No. AM7020, Thermo Fisher Scientific) and stored at −80 °C for gene expression analyses.

Supplementary files can be found online, including the step-by-step protocol for the mRNA analysis.^24^^,^^25^

### Histological analysis

The biopsies were embedded into paraffin using the following laboratory protocol. The biopsies were fixated in 4% formalin solution. After fixation, formalin was washed out and replaced by increasing concentrations of ethanol. The tissues were then embedded in paraffin and cut into 5 μm-thick sections using a rotary microtome (Leicah) at a clearance angle of 5°. Ribbons of tissue were placed on the surface of a heated water bath with demineralised water. Sections were fit on the Silane-coated (VWR) slides, and heat fixed in a heating chamber for ≥24 h at 37 °C. Afterwards, samples were rehydrated using a 100% > 90% > 70% ethanol gradient and submersed into Haematoxylin for 5 min, followed by 5 min under indirect running tap water. Samples were differentiated using 1% ethyl alcohol for 5 s and washed under indirect running water for 1 min. Samples were finally submersed in 1% Eosin for 3 min and washed under indirect running tap water for 1 min. Haematoxylin and Eosin (H&E) stained samples were covered by rectangular cover slips (VWR).

Following H&E staining, the slides were digitised, and point counting was performed as provided within the Medical Image Manager (MIM) software (Version 0.99 by HeteroGenius Ltd. UK). First, the tissue piece was manually outlined. Subsequently, 300 measurement points were placed into the outline using random systematic sampling (example in Supplementary Figure S2a). Each point was manually reviewed and classified as either connective tissue, adipose tissue, inflammation, blood cells, muscle fibres, other, or non-informative (point placed outside tissue). The point categorisation was performed blindly by an observer after appropriate training and independently verified by a pathologist. Difficult cases were discussed jointly. The relative number of points per tissue type per slide was calculated and compared between groups. Furthermore, adhesion slides were scored for the presence of foreign body material, which was observed by visualisation.

Immunohistochemical (IHC) staining was performed on 2 μm sections on a Ventana BenchMark ULTRA autostainer using the DAB Opti View IHC Detection Kit (Roche, Mannheim, Germany). Primary antibodies against S100 (ready to use (RTU); clone 4C4.9; Roche; RRID: AB_3676362), calretinin (RTU; clone SP65; Roche; RRID: AB_3676363), or synaptophysin (1:100; clone MRQ-40; Cell Marque; RRID: AB_3096182) were used to identify nerve fibres. A step-by-step protocol for the IHC markers S-100, Calretinin, and Synaptophysin is added in the Supplementary material (Supplementary IHC Protocol). In summary, the formalin-fixed, paraffin-embedded tissue sections were heated in an oven at 60 °C for 4 min, followed by deparaffinisation. Pre-primary peroxidase activity was blocked before incubation with the primary antibody for 20 min. Optiview HQ linker and OptiView HQ Universal Linker were applied, each incubating for 8 min. Detection was performed using the OptiView HRP Multimer system. Subsequently, the tissue was counterstained by Haematoxylin, and post-counterstained by bluing reagent. All slides with stained tissue samples were digitised at 40× magnification using an Aperio T2 scanner (Leica Microsystems). For IHC staining a colour threshold was determined for each marker to distinguish between positive and negative staining, which was quality controlled visually. The proportion of immunoreactive positive surface area to the total tissue area was quantified using MIM (represented in Supplementary Figure S2b, and view mode in Supplementary Figure S2c) and expressed as parts per million (ppm). The quantity of nerve fibres divided by the total tissue area was defined as nerve density.^26^

### Real time quantitative polymerase chain reaction (RT-qPCR)

**Fig. 1:**
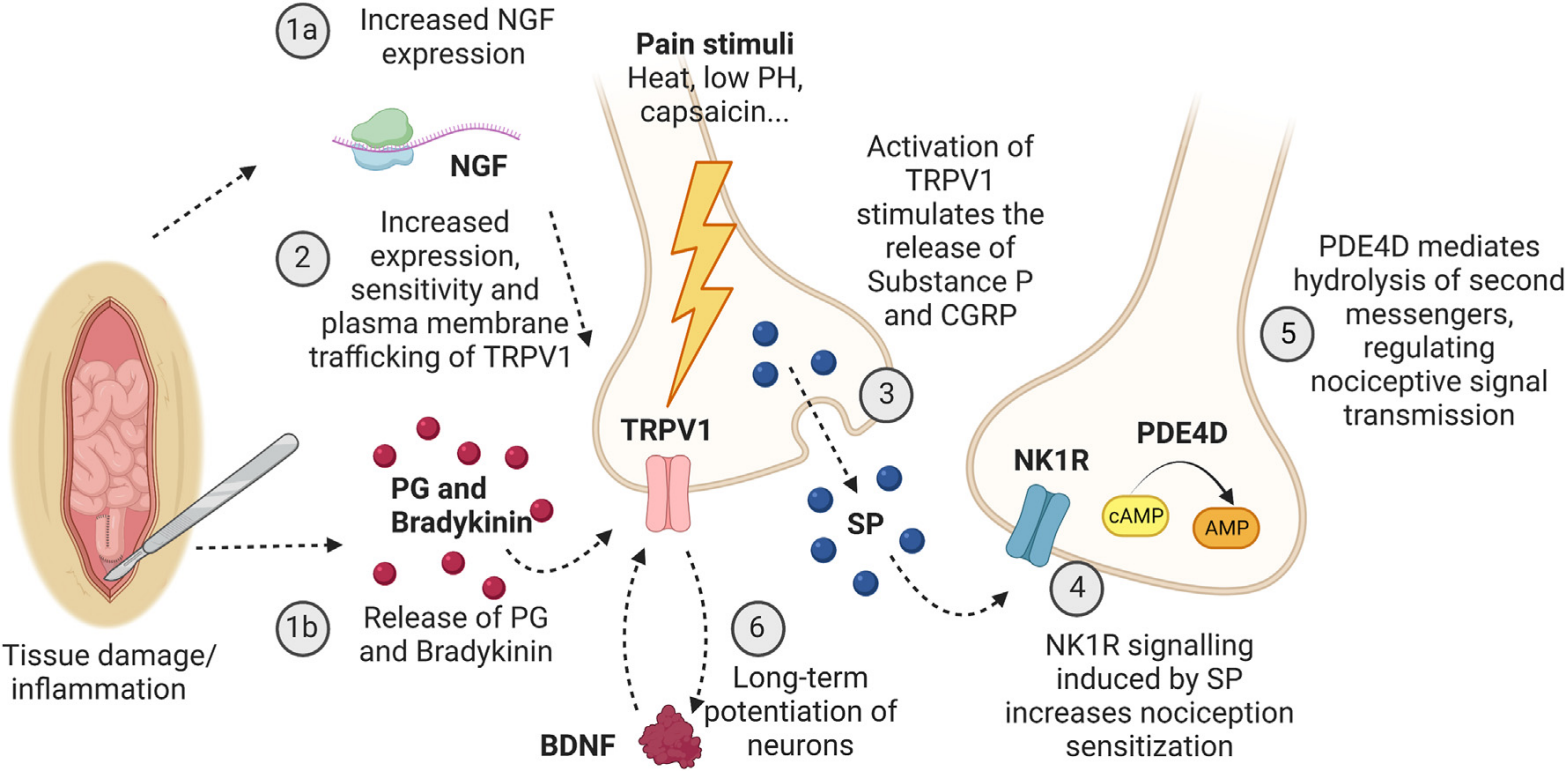
Pathway involved in nociceptive signalling triggered by tissue damage (created with BioRender.com). Following tissue damage or inflammation, both nerve growth factor (NGF) expression is increased (1a) and prostaglandin (PG) and bradykinin are released (1b). Both pathways (1a+1b) increase expression, sensitivity and plasma membrane trafficking of TRPV1 (2), triggering the long-term potentiation of neurons such as brain-derived neurotrophic factor (BDNF). Activation of transient receptor potential vanilloid 1 (TRPV1) stimulates the release of substance P (SP) and calcitonin gene-related peptide (CGRP) (4). SP induces neurokinin 1 receptor (NK1R) signalling, increasing nociception sensitisation (5). NGF, Nerve Growth Factor; PG, Prostaglandin; TRPV1, Transient receptor potential vanilloid 1; BDNF, Brain-derived neurotrophic factor; SP, Substance P; NK1R, neurokinin 1 receptor.

The level of mRNA expression of five candidate genes was assessed: *TRPV1*, tachykinin precursor 1 (*TAC1*) encoding for SP, tachykinin receptor 1 (*TACR1*) encoding for NK1R, *NGF*, and *BDNF* (Fig. 1). The biopsies of adhesions submerged in RNAlater were stored at −80 °C after surgery.

For the RNA isolation, RNAlater solution was removed from the adhesion samples, and a maximum of 8 mg of tissue was excised and homogenised to a powder with liquid nitrogen in a mortar. Total RNA was isolated using Qiagen RNeasy micro kit (Cat. No. 79256, Qiagen) following the manufacturer's protocol, combined with 10 min 0.22 mg/ml proteinase K (Qiagen, Cat. No. 19131) treatment at 55 °C after lysis and on-column DNase treatment of 15 min at room temperature. The concentrations of RNA were determined using the NanoDrop 2000c Spectrophotometer.

cDNA was synthesised using the iScript cDNA Syntheses Kit (cat. No. 1708891, BioRAD) according to manufacturer's instructions, with 350 ng of RNA as input. The cDNA was diluted 5 times and stored at −20 °C. For RT-qPCR, 8.75 ng template (2.5 μl) was used per well (assuming 1:1 conversion), along with 2 × 6.25 μl Power SYBR™ Green Master Mix (ref. nr. A25742, Thermo Fisher Scientific) and 0.5 μM of forward and reverse primer. A total reaction volume of 12.5 μl was manually pipetted in triplicate for each gene–target combination into a Hardshell PCR plate (art. No. HSP-9601, Bio-Rad). Amplification cycles were performed using a PCR machine (CFX Connect™ Real-Time PCR Detection System, model No. 1855200), with the following conditions: 7 min at 95 °C for denaturation of cDNA followed by 40 amplification cycles of 15 s at 95 °C and annealing for 1 min at 60 °C. Finally, a melt curve was acquired. A more detailed protocol for the analysis of mRNA expression can be found in the Supplementary information (Supplementary Information Protocol Pain Pad). Primer sequences and primer validation properties can be found in the Supplementary Table S1.

If the cycle threshold (Ct) value exceeded 35 or was N/A (non-applicable), it was considered a non-detectable value. The adhesion sample was categorised as valid non-detectable when expression of housekeeping genes but not from target genes was found. The biopsies with non-detectable expression levels of both the housekeeping genes and target genes were considered low RNA biopsies and excluded from further analyses.

### Statistics

In some patients the amount of adhesive tissue available for biopsy was very limited, thus limiting their histological and molecular analyses. In cases of missing data, cases were excluded per analysis.

We aimed to be able to detect a 50% increase in RNA expression of the genes of interest with 90% power. For 90% power, 23 patients were required to detect a statistically significant difference with two-sided p < 0.05. Taking potential losses into account, we therefore aimed at including 30 patients per group.

Baseline characteristics of the two groups (chronic pain and controls) were evaluated using Mann–Whitney U Test for continuous variables, and Chi–Square test for categorical variables. Peritoneal adhesion index (PAI) was scored by the surgeon and was calculated based on the adhesion scores per area. Adhesions were scored as filmy, blunt dissection possible (1 point); strong, sharp dissection necessary (2 points); very strong, requiring sharp dissection and damage hardly preventable (3 points).^27^

For patients with multiple biopsies, morphological features and molecular expression results were averaged per patient to facilitate comparisons. In patients with both target and non-target site biopsies, the values were average per type of biopsy separately.

Comparison between the chronic pain and control group was conducted for H&E and IHC stained sections using an independent t-test as the result values exhibited normal distribution after logarithmic transformation.

Subgroup analysis was performed to assess the relationship between the proportion of nerve fibres in the IHC slides and the proportion of tissue type, utilising Pearson's correlation, after visual inspection of linearity in the scatterplots. A two-sided p-value of <0.05 was considered statistically significant. Detailed description on the interpretation of Pearson's correlation results have been described previously.^28^ Analyses of histology and IHC results, including scatterplots, were made using IBM SPSS Statistics 29.

Final quantitative analysis of gene expression was conducted according to Hellemans et al., using qBase+ (v3.4) software [3].^29^ This software has an advantage over the traditional Livak (2-ΔΔCt) method as it accounts for primer-specific amplification efficiencies and allows for multi-gene normalisation [3, 4]. RT-qPCR data were expressed as mean expression ratios ± standard deviation (SD), normalised to the housekeeping genes. To assess statistical significance between experimental groups, a one-way analysis of variance (ANOVA) was performed on the log2-transformed data using GraphPad Prism (version 8.4.2). Post-hoc comparisons were conducted using Dunnett's multiple comparisons test. Gene expression levels were compared between the two groups by a two tailed T-test.

Subgroup analyses were conducted to assess variations in histology, IHC, detection rate, and expression levels of the target genes. Results from patients with adhesions collected from a designated target area, patients with adhesions from a non-target area, and patients experiencing diffuse pain were compared to those of the control group. Furthermore, an intrapatient analysis was performed to evaluate histology, detection rate and expression levels of the target genes in patients with adhesions from both target and non-target area to identify potential differences within patients. For IHC analysis, only target area biopsies were used if multiple biopsies were available, due to limited marker availability.

A p-value of less than 0.05 was considered statistically significant. Given the exploratory nature of the study, no additional correction for multiple testing was applied. This study followed the STrengthening the Reporting of OBservational studies in Epidemiology (STROBE) reporting guidelines.

### Role of funders

This study was funded by a grant from The Dutch Governmental Organisation for Health Research and Development (ZonMw): ’A New View of Chronic Pain from Adhesions’, but the funder did not have any role in study design, data collection, data analyses, interpretation, or writing of the report.

## Results

**Fig. 2:**
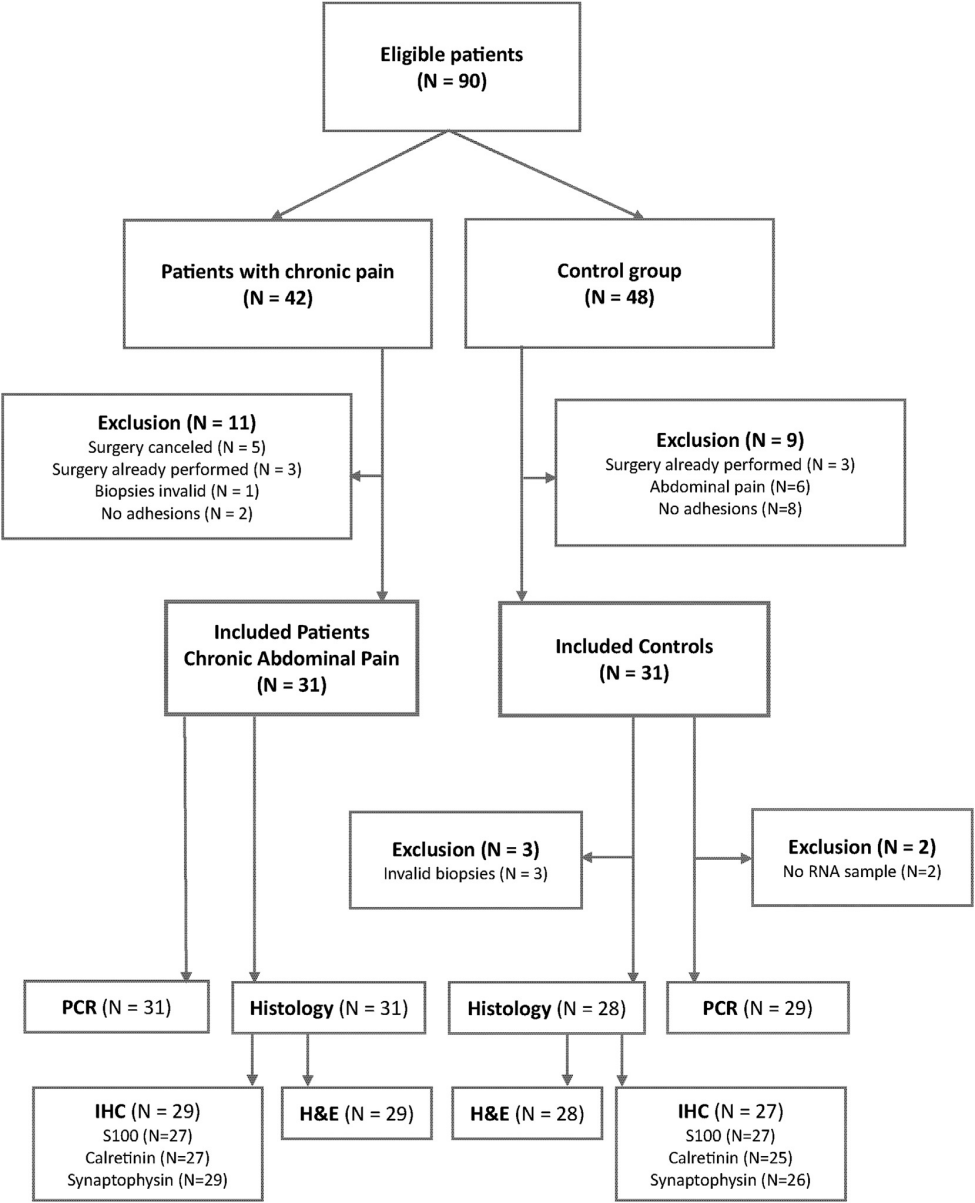
Flowchart of included patients (n = 62) with reasons for exclusions (n = 28). PCR, RT-qPCR; H&E, Haematoxylin and Eosin; IHC, immunohistochemistry.

A total of 90 eligible patients provided consent for inclusion in this study, with 28 individuals subsequently excluded (reasons specified in the flowchart in Fig. 2). This resulted in 62 inclusions, 31 patients with chronic abdominal pain and 31 patients in the control group. The median age was 57 years, ranging from 28 to 87 years. Females constituted the majority (43 of 62, 69%) of patients, as detailed in Table 1. No significant differences in baseline characteristics were observed between the patients with pain and controls. Patients were asked about their pain level pre-surgery and one year post-surgery. Almost half of the patients improved (15 out of 31, 48%), five reported an equal amount of pain (16%), and 11 patients deteriorated (35%).

**Table 1:** Baseline characteristics of all patients in the cohort, compared between patients with pain (n = 31) and controls (n = 31).

	Patients with pain n = 31	Controls n = 31	Total cohort n = 62	p value
**Sex**, n (%)				0.168
Female	24 (77%)	19 (61%)	43 (69%)	
Male	7 (23%)	12 (39%)	19 (31%)	
**Age category (years)**, n (%)				0.132
18–24	0	0	0	
25–44	11 (35%)	4 (13%)	15 (24%)	
45–64	13 (42%)	14 (45%)	27 (43%)	
65–84	7 (23%)	12 (39%)	19 (31%)	
≥85	0	1 (3%)	1 (2%)	
**Previous abdominal surgeries**, n (%)				0.294
1	4 (13%)	5 (16%)	9 (15%)	
2	7 (22%)	11 (35%)	18 (30%)	
3	4 (13%)	5 (16%)	9 (15%)	
4 or more	16 (52%)	8 (26%)	24 (40%)	
**Previous open surgeries**, n (%)				0.370
1	12 (39%)	17 (55%)	29 (47%)	
2	7 (23%)	6 (19%)	13 (21%)	
3	4 (13%)	2 (6%)	6 (10%)	
4 or more	6 (19%)	2 (6%)	8 (13%)	
**History of small bowel obstruction**, n (%)	10 (32%)	4 (13%)	14 (23%)	0.077
**Previous surgery categories**, n (%)				
Colorectal	16 (52%)	17 (57%)	33 (54%)	0.692
Gynaecological	15 (48%)	6 (20%)	21 (34%)	0.020
Appendectomy	11 (36%)	6 (20%)	17 (28%)	0.178
Abdominal wall	3 (10%)	8 (27%)	11 (18%)	0.084
Cholecystectomy	5 (16%)	6 (20%)	11 (18%)	0.694
HPB	1 (3%)	7 (23%)	8 (13%)	0.020
Upper GI	3 (10%)	2 (7%)	5 (8%)	0.668
Other	5 (16%)	1 (3%)	6 (10%)	0.093
**Preoperative medication use**, n (%)				
Paracetamol	9 (29%)	0	9 (15%)	0.001
NSAID	4 (13%)	0	4 (7%)	0.042
Opioids	7 (23%)	0	7 (11%)	0.006
Neuropathic analgesics	2 (6%)	1 (3%)	3 (5%)	0.573
**Malignancy**, n (%)	5 (16%)	12 (40%)	17 (28%)	0.038
**BMI**, median (range)	26.0 (16.0–36.9)	26.8 (19.5–33.7)	26.4 (16.0–36.9)	0.523
**Preoperative pain**, median (range)^a^				
Lower range	2.5 (0.5–6.0)			
Upper range	7.5 (2.5–9.5)			
Daily pain score	5.5 (1.0–8.0)			
**Peritoneal Adhesion Index (PAI)**, median (range)	6.5 (2–30)	5.5 (1–30)	6.0 (1–30)	0.150

(Continuous variables are analysed using Mann–Whitney U Test, categorical variables using Chi–Square test).

Upper GI: upper gastrointestinal HPB: hepatopancreatobiliary NSAID: Non-steroidal anti-inflammatory drugs BMI: Body mass index.

a Pain is scored according to the numeral rating scale (NRS) from 1 to 10.

### Adhesion morphology

**Fig. 3:**
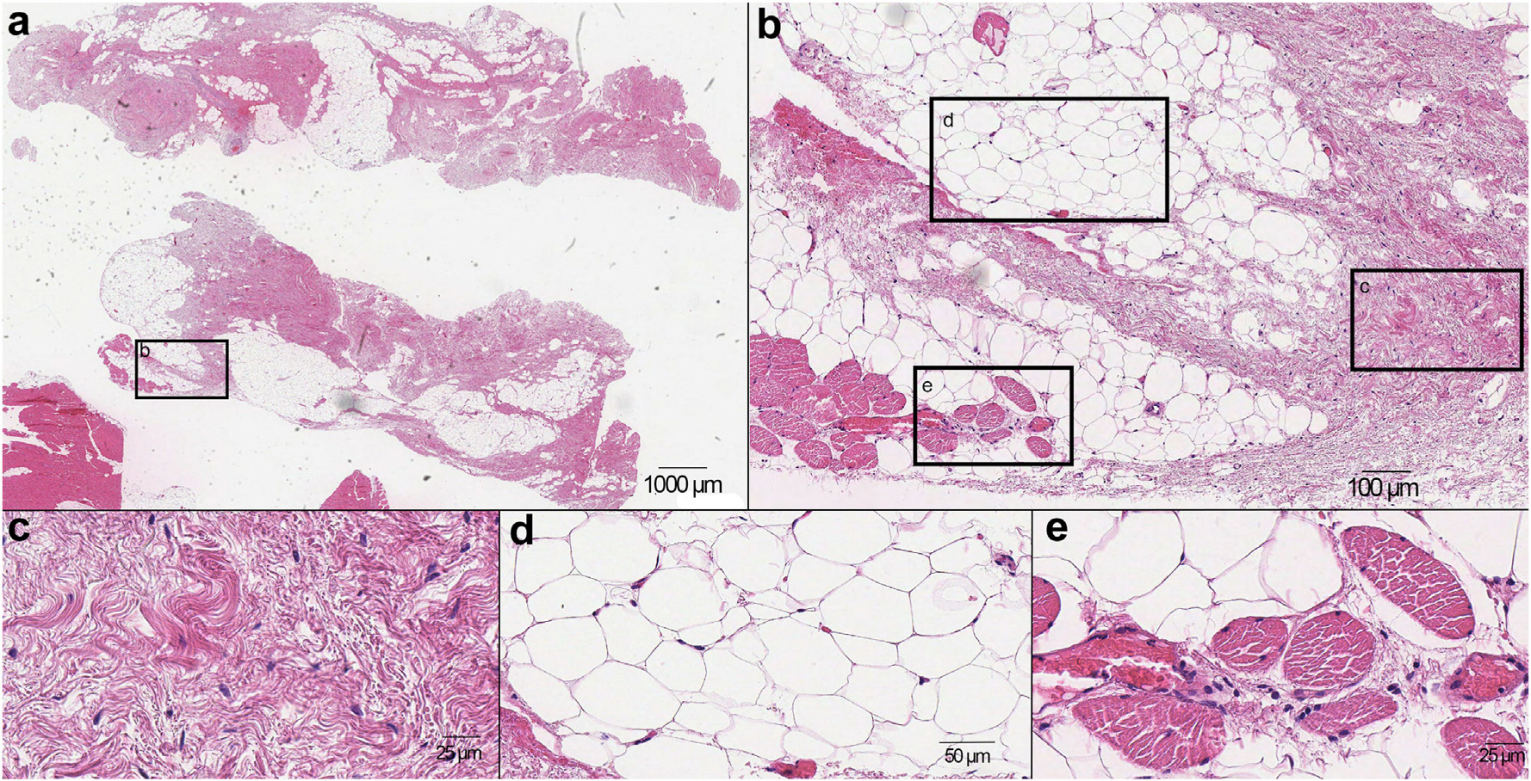
Representative image of the histology of an abdominal adhesion from one patient (digitised at 40× magnification using an Aperio T2 scanner (Leica Microsystems)) (a) Overview of a representative slide illustrating the predominant component of adhesion tissue; connective tissue (H&E staining) (b) Digital magnification of the left side of the lower adhesion (c) Digital magnification shows mature connective tissue with low cellularity (d) Digital magnification shows normal adipose tissue without inflammation (e) Digital magnification shows blood filled thin-walled vessels within the adipose tissue.

Adhesion tissue composition was assessed in H&E-stained sections. The adhesions comprised various tissue types, with the highest proportion being connective tissue (median 48.7%, range 6.8%–96.2%) and adipose tissue (median 37.9%, range 0–86.8%) (representative images in Fig. 3). Blood vessels accounted for a median of 4.5% (range 0–30.7%) of the total adhesion sample area of all patients (Supplementary Figure S3a and b). Foreign body material was present in 18 (30.5%) patients (Supplementary Figure S3c). The median (range) proportion of each tissue type, quantified by stereological point counting, is presented in Table 2. No significant differences in tissue composition were observed between patients with pain and controls.

**Table 2:** Histological characteristics of H&E-stained adhesion biopsies, the composition of adhesions are compared between patients with pain (n = 31) and controls (n = 28) using stereotypical point counting (independent t-test).

	Patients with pain (N = 31) median% (range)	Controls (N = 28) median% (range)	p value
Blood vessel	3.37% (0.00–12.50%)	5.70% (0.00–30.65%)	0.052
Muscle fibres	3.07% (0.00–29.76%)	9.96% (0.00–47.60%)	0.505
Connective tissue	48.72% (6.75–96.17%)	47.29% (9.86–94.56%)	0.908
Adipose tissue	41.77% (0.00–86.76%)	33.62% (0.00–86.27%)	0.378
Inflammation	0.77% (0.00–5.77%)	1.21% (0.00–8.61%)	0.440
Other	2.29% (0.00–67.88%)	2.21% (0.00–11.06%)	0.664

Category ‘Other’ = arrows indicating e.g., foreign material, air bubbles in the slides or non-clear tissue.

**Fig. 4:**
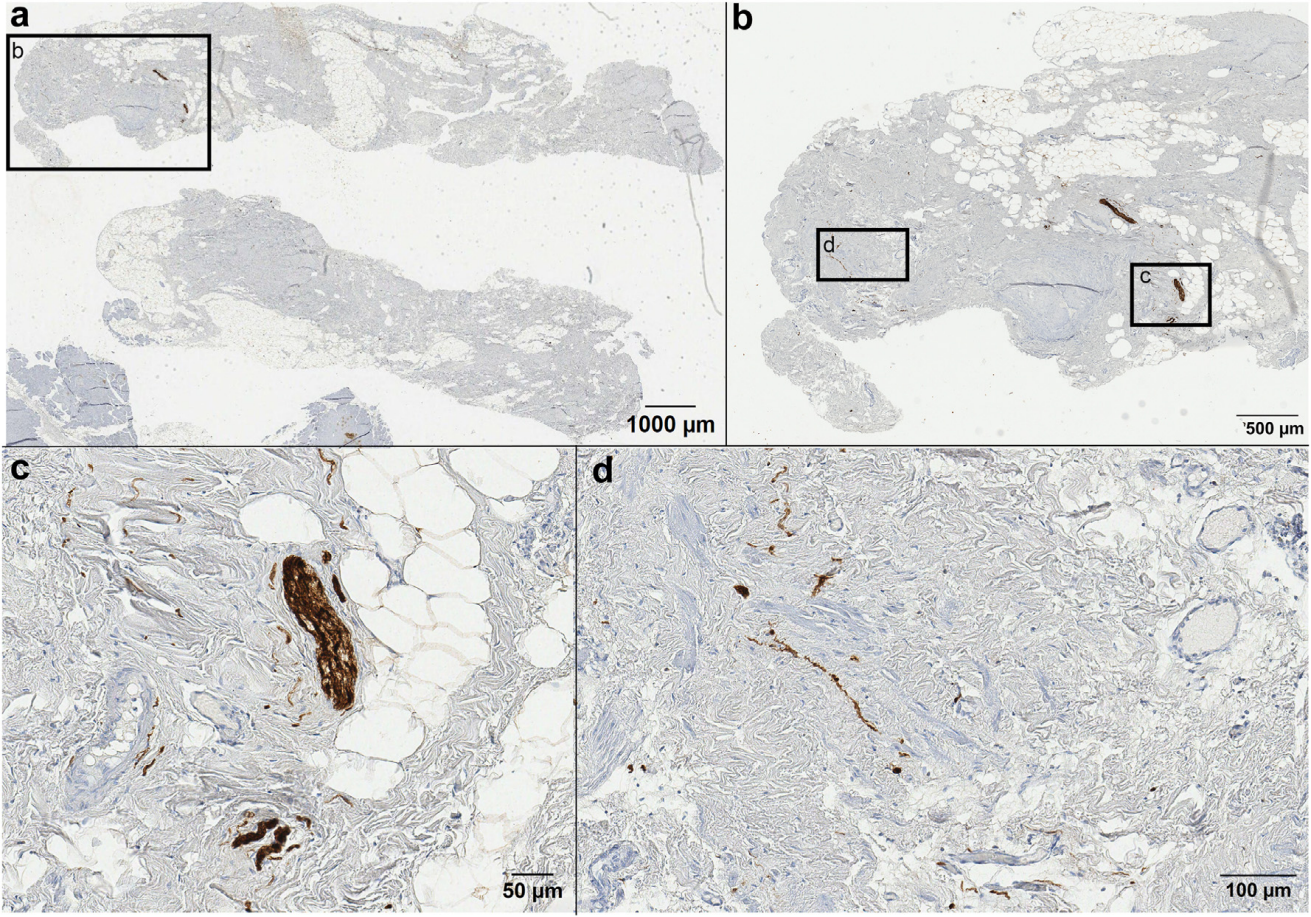
Representative image of the IHC of an abdominal adhesion from one patient (digitised at 40× magnification using an Aperio T2 scanner (Leica Microsystems)) (a) Adhesion biopsy stained with S100 (b) Digital magnification of the square of subfigure 4a (c) Digital magnification of subfigure 4b shows S100 positivity (brown) in a larger nerve bundle (d) Digital magnification of subfigure 4 b shows S100 positivity in thin nerve fibres.

**Fig. 5:**
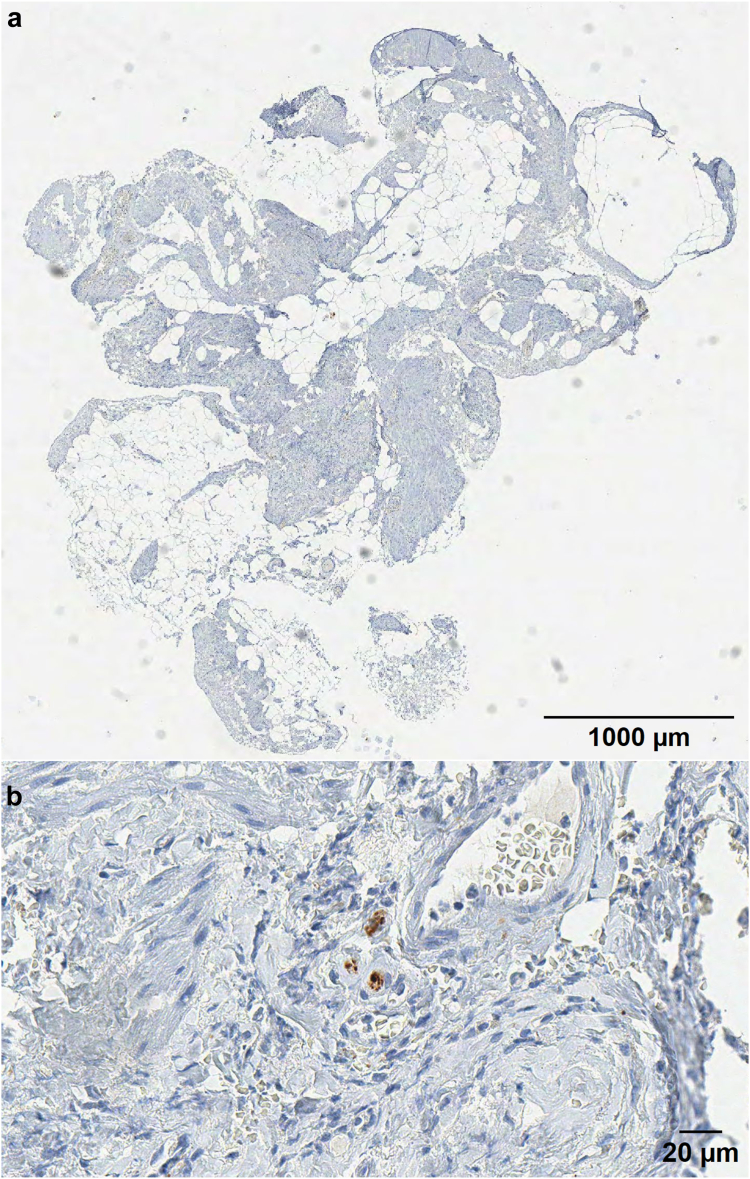
Representative image of the IHC of an abdominal adhesion from one patient (digitised at 40× magnification using an Aperio T2 scanner (Leica Microsystems)) (a) Representative adhesion biopsy stained with calretinin (b) Digital magnification of positive calretinin marked nerve fibres.

**Fig. 6:**
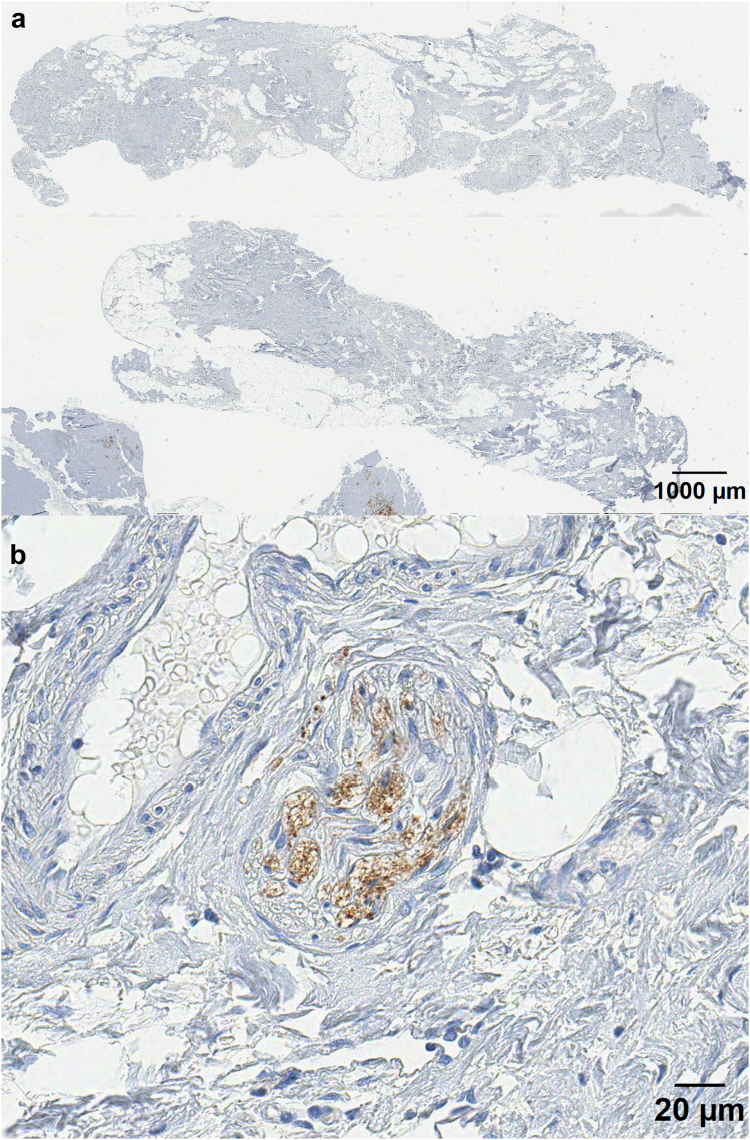
Representative image of the IHC of an abdominal adhesion stained with synaptophysin from one patient (digitised at 40× magnification using an Aperio T2 scanner (Leica Microsystems)) (a) Representative slide of an adhesion biopsy (b) Digital magnification shows positive marked nerve fibres.

IHC assessment of the adhesions showed positivity for S100, calretinin and synaptophysin in almost all sections from patients with pain and controls. Positively stained nerve fibres were either randomly distributed or present in a larger cluster, as illustrated in a representative slide of a S100 marked adhesion in Fig. 4. The detection threshold for positive staining was set at grey values between 19 and 87, within which the marker was recognised as positive and displayed in white in view mode. The median nerve density was significantly higher in adhesions from patients with pain compared to controls based on S100 IHC (597.0 ppm (Range 92.2–3223.2 ppm) vs 150.7 ppm (range 15.2–1683.8 ppm), p < 0.001 (independent t-test)) and calretinin IHC (462.6 ppm (range 72.7–2996.5 ppm) vs 274.6 ppm (range 35.3–3194.8 ppm), p = 0.040 (independent t-test)). Nerve density based on synaptophysin IHC showed no significant difference between the two groups (Table 3). Representative calretinin and synaptophysin slides can be found in Fig. 5, Fig. 6, respectively.

**Table 3:** Proportion of positive immunohistochemical markers indicating nerve fibres in adhesions, compared between patients with pain and controls (independent t-test).

	Patients with pain median% (range)		Controls median% (range)	p value
**S100**				
N = 27	597.0 ppm (92.2–3223.2 ppm)	N = 27	150.7 ppm (15.2–1683.8 ppm)	0.001^a^
**Calretinin**				
N = 27	462.6 ppm (72.7–2996.5 ppm)	N = 25	274.6 ppm (35.3–3194.8 ppm)	0.040^a^
**Synaptophysin**				
N = 29	677.7 ppm (84.5–6492.5 ppm)	N = 26	570.6 ppm (110.2–2040.3 ppm)	0.627

a p < 0.05.

A higher density of calretinin and/or synaptophysin-positive nerves correlated with a greater proportion of blood vessels in the adhesions of patients with pain (r = 0.525 and r = 0.660, respectively (Pearson's correlation)) (Table 4). Higher nerve density based on S100, calretinin, and synaptophysin IHC significantly and positively correlated (r = 0.414, r = 0.635, r = 0.530 respectively (Pearson's correlation)) with a higher proportion of connective tissue. Higher nerve density based on calretinin IHC and synaptophysin IHC significantly and negatively correlated with the proportion of adipose tissue (r = −0.618 and r = −0.575 respectively (Pearson's correlation)).

**Table 4:** Correlation between the proportion of adhesion histology tissue types and nerve density based on immunohistochemical marker (s100, calretinin, and synaptophysin) positivity in patients with pain and controls, analysed with Pearson's correlation.

	Patients with pain	p value	Controls	p value
	Correlation coefficient (r)		Correlation coefficient (r)	
	S100 (n = 27)		S100 (n = 27)	
Blood vessels	0.328	0.095	0.154	0.442
Muscle fibre	−0.180	0.368	−0.031	0.876
Connective tissue	0.414	0.032^a^	0.020	0.919
Adipose tissue	−0.011	0.956	−0.362	0.064
Inflammation	−0.160	0.424	0.026	0.896
Other	−0.258	0.194	−0.185	0.355
	**Calretinin (N = 27)**	**p value**	**Calretinin (N = 25)**	**p value**

Blood vessels	0.525	0.005^a^	−0.052	0.805
Muscle fibre	−0.021	0.919	−0.118	0.574
Connective tissue	0.635	<0.001^a^	−0.184	0.379
Adipose tissue	−0.618	<0.001^a^	0.254	0.221
Inflammation	0.079	0.696	−0.115	0.583
Other	−0.092	0.647	−0.075	0.720
	**Synaptophysin (N = 29)**	**p value**	**Synaptophysin (N = 26)**	**p value**

Blood vessels	0.660	<0.001^a^	−0.039	0.849
Muscle fibre	0.101	0.604	−0.070	0.734
Connective tissue	0.530	0.003^a^	0.157	0.443
Adipose tissue	−0.575	<0.001^a^	−0.061	0.766
Inflammation	0.124	0.523	−0.121	0.556
Other	−0.146	0.450	−0.320	0.111

a p < 0.05.

Nerve density was compared in a subgroup analysis, revealing no significant difference in the composition of adhesions between the subgroups (Supplementary Table S2). Furthermore, nerve density did not exhibit a significant difference between the subgroups, as indicated in Supplementary Table S3.

The intrapatient analysis similarly showed no differences in the adhesion composition between sites associated with pain and sites not associated with pain (Supplementary Table S4).

### Gene expression in adhesions

**Fig. 7:**
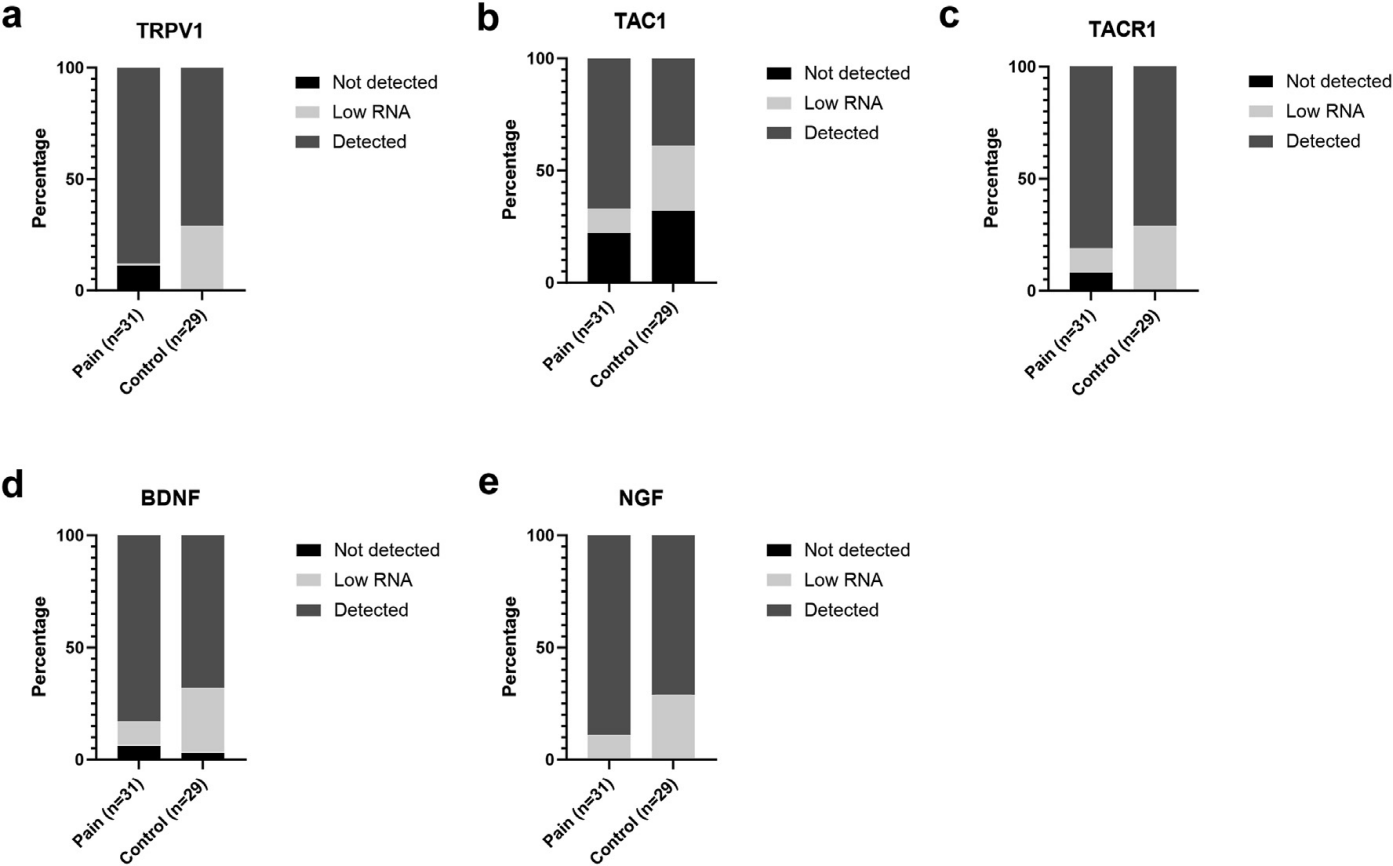
Detection rate of target genes using RT-qPCR comparing patients with pain (n = 31) and controls (n = 29). Y-axis displays the percentage of patients., and the groups are displayed on the X-axis a) Detection rate of transient receptor potential vanilloid 1 (TRPV1) in patients with pain and controls b) Detection rate of tachykinin precursor 1 (*TAC1*) encoding for substance P(SP) in patients with pain and controls c) Detection rate of tachykinin receptor 1 (*TACR1*) encoding for neurokinin 1 receptor (NK1R) in patients with pain and controls d) Detection rate of Brain-derived neurotrophic factor (*BDNF*) in patients with pain and controls e) Detection rate of nerve growth factor (*NGF*) in patients with pain and controls.

The baseline characteristics of the patients for whom gene expression could be analysed are shown in Supplementary Table S5. There were significantly more females in the group with pain (23 of 29, 79%) compared to the control group (12 of 23, 52%; p = 0.022 (Chi–Square test)). The gene expression levels of *TRPV1*, *TAC1*, *TACR1*, *BDNF*, and *NGF* were quantified using RT-qPCR. In two patients with pain and six control patients, RNA expression of the housekeeping genes was unmeasurable, possibly due to insufficient mRNA, resulting in the exclusion of these samples. The final analysis included 29 patients with pain and 23 control patients. The gene detection rates for *TRPV1*, *TAC1*, *TACR1*, *BDNF*, and *NGF* are depicted in Fig. 7, *NGF* showed the highest detection rate among all genes and was identified in all samples with sufficient RNA. Adhesions in patients with pain did not exhibit a significant difference in detection rates for all genes compared to controls.

**Fig. 8:**
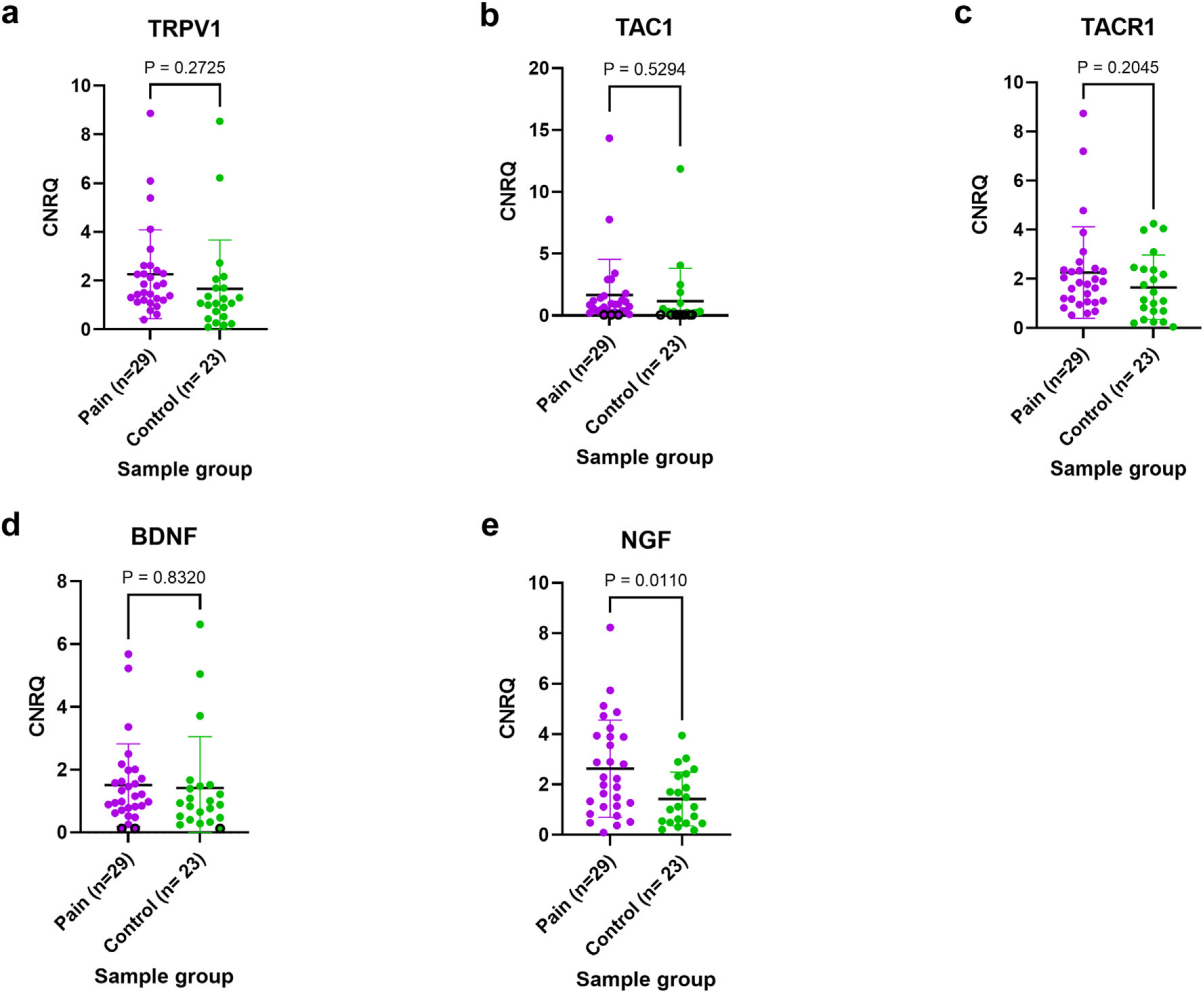
Expression levels of the studied genes, comparing adhesions from patients with pain (n = 29) with controls (n = 23) by RT-qPCR. (a) Expression levels of transient receptor potential vanilloid 1 (TRPV1) in patients with pain and controls, p = 0.273 (two-tailed t-test) (b) Expression levels of tachykinin precursor 1 (*TAC1*) encoding for substance P (SP) in patients with pain and controls, p = 0.529 (two-tailed t-test) (c) Expression levels of tachykinin receptor 1 (*TACR1*) encoding for neurokinin 1 receptor (NK1R) in patients with pain and controls, p = 0.205 (two-tailed t-test) (d) Expression levels of brain-derived neurotrophic factor (*BDNF*) in patients with pain and controls, p = 0.832 (two-tailed t-test) (e) Expression levels of nerve growth factor (*NGF*) in patients with pain and controls, p = 0.011 (two-tailed t-test). CNRQ, Calibrated and normalised relative gene expression.

The comparison between gene expression levels in patients with pain and controls is illustrated in Fig. 8. *NGF* expression was significantly higher (p = 0.0110 (two-tailed t-test)) in patients with pain compared to controls. However, no differences were observed for *TRPV1*, *TAC1*, *TACR1*, or *BDNF expression*.

In the subgroup analysis comparing adhesions from the target area, non-target area, and diffuse pain in patients with pain to controls, no significant differences were found in the detection rate of the genes (Supplementary Figure S4). Adhesions from the target area in patients with pain exhibited significantly higher gene expression of *NGF* compared to controls. (Supplementary Figure S5. *TACR1* (encoding for NK1R) was significantly higher in patients with diffuse pain compared to all other groups.

Furthermore, the intrapatient subanalysis revealed no significant differences in detection rates (Supplementary Figure S6) or expression levels (Supplementary Figure S7) between biopsies from target and non-target areas.

## Discussion

This study aimed to assess the morphological composition and RNA expression of adhesions in patients with chronic abdominal pain in comparison to those without pain. The quantitative histological analysis revealed that adhesions predominantly comprise connective and adipose tissues. Utilising IHC, we demonstrated a two to four times higher nerve density in adhesions of patients with chronic abdominal pain compared to the control group. Additionally, using quantitative RT-qPCR, we observed a significant increase in the gene expression of *NGF* in patients with pain. These findings suggest potential structural differences in the development of adhesions between patients with and without chronic pain. The expression of components in the TRPV1-SP-NK1R pathway was detected in most patients; however, no significant differences were noted between the two groups.

The observation that adhesions predominantly consist of connective and adipose tissue aligns with previous literature.^12^ The presence of blood vessels seen in adhesions is interpreted as a consequence of the inflammatory response initiated by tissue damage during surgery, a phenomenon known to promote angiogenesis.^30^^,^^31^

In our study, the increased nerve density and concomitant increase in *NGF* expression levels observed in adhesions of patients with pain supports the notion of nerve ingrowth in adhesions as an explanation for adhesion-related pain. NGF protein plays a crucial role in normal tissue healing by promoting the growth and survival of peripheral sensory nerves.^32^ Elevated *NGF* expression levels contribute to long-term nociceptive hypersensitivity by influencing the activity and/or expression of channels and receptors.^33^ Furthermore, NGF is suspected to increase nerve terminal density in peripheral tissues through local neuronal sprouting.^33^

This study demonstrates that patients with adhesion-related pain exhibit increased nerve density and elevated RNA expression levels of *NGF* compared to those without pain. While previous studies have demonstrated the presence of nerve tissue in human adhesions from patients with various complaints, this study conducts a comparative quantitative assessment of nerve density in adhesions from patients with pain and without pain.^12^^,^^34^ Two previous histological studies compared adhesions from patients with and without pain, with one solely focussing on the prevalence of nerve tissue and finding no significant difference in the histological (H&E) and IHC analysis between the groups.^35^ Another study assessed nerve fibres in adhesions using a semiquantitative scale (rare, few, many).^34^ Both studies lacked a detailed description of included patients, including diagnostic criteria for adhesion-related pain, and analyses were qualitative or at most semiquantitative.^34^^,^^35^ Furthermore, their findings were not substantiated by molecular analyses.

A strength of our study is the assessment of nerve density using three distinct markers, each highlighting different aspects of nerve fibres. S100, a highly sensitive marker, was explored to label myelinated nerve fibres, albeit with the potential of marking adipose cells positively.^36^, ^37^, ^38^, ^39^ To avoid adipose tissue inclusion, a trained researcher meticulously selected areas for positive S100 analysis. The calretinin marker, known for identifying ganglion cells and nerve fibres, boasts a specificity for nerve tissue exceeding 93% in prior literature.^40^ Calretinin plays a pivotal role in transporting calcium in nerve fibres, thereby modulating neuronal excitability. In conclusion both highly sensitive and specific markers for nerve fibres were found to be higher in patients with pain compared to controls, indicating a genuine increase in nerve fibre density.

Synaptophysin, which visualises presynaptic vesicle protein involved in neurotransmitter release, demonstrated no difference between nerve density between patients with pain and controls in our study.^41^ Although this marker was positive in the majority of patients, implying synaptic transmission in adhesions, the absence of a difference between patients with pain and controls suggests that this function may not be the explanation for pain related to adhesions.

Increased nerve density in patients experiencing chronic pain associated with adhesions could suggest a neuropathic component in pain related to adhesions. Studying the neuropathic aspect of pain associated with adhesions holds promise for future studies, e.g., utilising the DETECT pain questionnaire to distinguish complaints.^42^ Presently, the only evidenced based treatment for chronic adhesion-related pain involves adhesiolysis with the application of an adhesion barrier.^8^ However, a significant portion of patients is not eligible for this treatment due to various surgical and medical reasons, necessitating reliance on conservative treatment. Despite the absence of a standardised analgesic regimen for this specific chronic pain, conservative treatment primarily relies on analgesia. One pilot study showed promising results with pregabalin treatment, revealing a 2-point decrease (on a 10-point scale) in pain after a seven-week treatment period compared to the placebo group.^43^ This trial supports the hypothesis that adhesion-related pain might have neuropathic components, suggesting potential benefits from treatment with neuropathic pain medication.

Although an extensive body of literature describes molecular mechanisms leading to adhesion development in animal models, those studies primarily focus on the acute phase and do not include measurements of *NGF* gene expression.^19^^,^^20^^,^^44^^,^^45^ Therefore, another strength of this study is addressing this gap in the literature. To comprehend adhesion-related chronic pain, insights into the matured phase of adhesions are crucial. This study describes RNA expression patterns in matured human adhesions. Our hypothesis is that the sustained high expression levels of NGF, as a part of continued tissue remodelling, play a role in the development of pain related to adhesions.

Despite observing no significant differences in the RNA expression levels of the TRPV1-SP-NK1R axis between control patients and patients with pain. This axis seemed of particular interest, as it has been shown to be important both in the acute phase of adhesion formations as well as being involved in many chronic pain conditions.^46^^,^^47^ Despite the lack of difference in expression levels, the high detection rate could still indicate the potential involvement of the axis in the pathophysiology of pain. The TRPV1-SP-NK1R pathway is expressed in nerve tissue (Fig. 1). However, genes of the TRPV1-SP-NK1R axis are mainly expressed in the nerve cell body, and transcribed to proteins which then migrate to the axon terminal.^46^^,^^47^ It is possible that the nerve fibres in adhesions are mainly composed of axon terminals, which might explain the lack of difference in RNA expression levels between the two groups in our cohort. Future proteomic studies could add further to the understanding of the TRPV1-SP-NK1R axis in adhesion-related pain.^46^^,^^48^ The axis is of clinical interest because resolvins, which are able to modulate TRPV1 activation, are being developed as a new class of analgesic agents.^49^ Future larger replication studies might find a difference, both in RT-qPCR measurements and in expression of these molecules by IHC and proteomics.

In our study we found a strong female predominance in patients included, which aligns with previous research on adhesion-related pain.^8^ However, to date it is unclear whether women truly develop chronic pain more often after abdominal surgery or that these differences are caused different pain behaviour and seeking medical help for pain. The ongoing prospective PainTrac study on development of chronic pain after surgery intends to answer these question (Clinicaltrials.gov NCT04088838). If there is a real difference in incidence of chronic pain sex hormones might also have a role in the development of the different adhesion phenotypes that result in pain.

One limitation of this study is the challenge of diagnosing adhesion-related pain. Adhesion-related chronic abdominal pain lacks a typical clinical presentation, with symptoms varying based on adhesion location and involved organs. In our study only patients with daily continuous or intermittent pain were included, while patients with episodes of pain from ASBO were excluded. Adhesions are commonly found after surgery, potentially leading to the false attribution of chronic pain to adhesions. However, falsely attributing pain to adhesions would not invalidate the correlations found comparing morphological and molecular characteristics in adhesions between patients with pain and without pain. Perfect matching of patients with pain and controls was also not possible due to the prospective nature of the study, and the need to take biopsies prospectively. Nevertheless, only small differences were found.

We analysed the smaller groups on some of the baseline characteristics. In the subgroup analysed by RT-qPCR for gene expression, samples that couldn't be used were mainly from males. Therefore, there were significantly more females left in the pain group compared to the control. Furthermore, we do not have data on race or lifestyle behaviour. This is an interesting topic for future research into generalisability of results to different ethnic groups.

Foreign material was found in 18 of 59 (30.5%) H&E-stained patient biopsies, probably originating from previous surgery which formed the adhesions. These patients were mainly controls. In the subanalysis the pain group is split up in subgroups according to area, and the controls are used as a comparison. Therefore, the small ‘other’ category turns out to be statistically significantly different. 0% compared to 1.5% is significant in this analysis, but not clinically relevant.

In this study we compared histological and molecular features of adhesions between patients with pain and without pain. We did not assess in multivariate the contribution of other patient characteristics on adhesion phenotype. Given the relatively small sample size this was not statically feasible. Pain is considered the clinically relevant outcome of adhesions. Potentially, there is a risk for residual confounding, e.g., time elapsed between the previous surgery and the taking of surgery biopsies, preoperative medication use, malignancy and other non-studied factors. However, this study has an observational design, providing important new insights. With these insights, topics for new studies are presented. In our study, the subgroup analyses were small, and larger studies are required to verify these relations.

The findings of our study also raise new questions on the origin and type of nerve fibres and pain in patients with adhesion-related pain. We did not differentiate between biopsies of adhesions to the abdominal wall and adhesions between viscera, nor were we able to detect if nerve fibres originated from the peritoneal or visceral slide. Possibly, the origin of the nerve fibres also impacts pain symptoms and the type of pain, i.e., visceral or nociceptive pain.

The RNA yield from adhesion tissue biopsies limits the analysis potential, allowing for RT-qPCR analysis of only a limited number of genes. For this study, genes most likely associated with pain and adhesions were selected based on current literature. Future studies could explore other potentially involved cascades. Caspases are of potential interest as they also seem to have a role in adhesion formation and have previously been linked to development of chronic pain.^50^^,^^51^ Furthermore, patients with chronic adhesion-related pain often resort to surgical treatment involving adhesiolysis and the application of an anti-adhesive agent, improving the quality of life in up to 80% of patients.^8^ However, whether the outcome of adhesiolysis is associated with the phenotype of adhesions in these patients remains to be determined.

This study revealed increased nerve density in adhesions from patients with chronic abdominal postoperative pain compared to those without pain. The correlation between nerve density and amount of blood vessels, connective tissue and adipose tissue, suggests a difference in the morphological composition as a potential explanation for adhesion-related pain. Increased *NGF* expression may indicate an altered adhesion formation process in patients with chronic abdominal pain. Overall, the findings of this explorative study contribute to unravelling the mechanisms of pain related to post-operative abdominal adhesions.

## Contributors

Masja Karina Toneman, M.D. (Conceptualisation; Data curation; Formal analysis; Investigation; Methodology; Project administration; Visualisation; Writing—original draft; Writing—review & editing).

Priscilla Pauline Marianne Faas, Msc (Data curation; Formal analysis; Investigation; Resources; Writing—original draft; Writing—review & editing).

Maurits Johan Christian Antoine Marie Gielen, Msc (Data curation; Formal analysis; Project administration; Resources; Writing—original draft; Writing—review & editing).

Valentina Carotti, Msc (Formal analysis; Investigation; Methodology; Resources; Writing—review & editing).

Anne Elisa Adriana van Oirschot, Bsc (Data curation; Formal analysis; Resources; Writing—review & editing).

Judith Petronella Maria Mangnus, Bsc (Data curation; Formal analysis; Investigation; Writing—review & editing).

Anne Wintjens, MD (Conceptualisation; Investigation; Methodology; Writing—review & editing) Casper Mihl, MD PhD (Conceptualisation; Methodology; Supervision; Writing—review & editing) Josephine Huige, MD (Conceptualisation; Methodology; Writing—review & editing).

Bianca Grosser, MD (Formal analysis; Resources; Writing—review & editing).

Marjolein Blussé van Oud-Alblas, MD (Data curation; Resources; Writing—review & editing).

Koen Willem van Dongen, MD PhD (Data curation; Resources; Writing—review & editing).

Nicole Dorine Bouvy, MD Prof (Conceptualisation; Methodology; Supervision; Visualisation; Writing—review & editing).

Harry van Goor, MD Prof (Conceptualisation; Methodology; Visualisation; Writing—review & editing) Daniel Keszthelyi, MD Prof (Conceptualisation; Methodology; Resources; Writing—review & editing) Heike Irmgard Grabsch, MD PhD (Conceptualisation; Formal analysis; Investigation; Methodology; Supervision; Writing—review & editing).

Richard Peter Gerardus ten Broek, MD PhD (Conceptualisation; Formal analysis; Funding acquisition; Methodology; Resources; Supervision; Visualisation; Writing—review & editing.

All authors read and approved the final version of the manuscript.

M.K.T, A.E.A. vO, J.P.M. M, and R.P.G. tB accessed and verified the underlying data.

## Data sharing statement

The data that support the findings of this study are stored in the Radboud Data Repository and are available upon reasonable request.

## Declaration of interests

Richard Petrus Gerardus ten Broek received an unrestricted research grant (Investigator initiated) for an unrelated study from Temple Pharmaceutical, and travel grant as speaker for the Adhesions Improvement Summit in Washington DC, by scientific society of the American College of Surgeons (all paid to host institute).

Daniel Keszthelyi has received grants from The Dutch Governmental Organisation for Health Research and Development (ZonMw), Maag Lever Darm Stichting (MLDS), Rome Foundation, Horizon Europe, Horizon 2020, UEG, all unrelated to current work. Further, he is in the data safety monitor board (DSMB) for a probiotics trial (payment made to host institution), he is associate editor for United European Gastroenterology (UEG) Journal (payment to host institute), multimedia Editor for Neurogastroenterology and Motility (payment to host institute), and chair of the Neurogastroenterology section of the Dutch Society of Gastroenterology (unpaid) Steering Committee member of the European Society for Neurogastroenterology (ESNM) (unpaid).

Heike Irmgard Grabsch received charity from the Cancer Research UK (not related to this manuscript), charity form Hanarth Fonds NL (not related to this manuscript), and UK government funding from the British Research Council (not related to this manuscript). Further, she received royalties for contribution of a book chapter. She is an unpaid board member of Pathological Society of Great Britain and Ireland, and unpaid council member of the international Gastric Cancer Association.

## Appendix A Supplementary data

Supplementary IHC Protocol

Supplementary Information Protocol Pain Pad
