# Supplementary material for: Morphological features and molecular mechanisms in peritoneal adhesions from patients with chronic abdominal postoperative pain
Source: eBioMedicine. 2025 May 23;116:105746. doi: 10.1016/j.ebiom.2025.105746 (PMC12153383; doi:10.1016/j.ebiom.2025.105746)
Supplement: Supplementary IHC Protocol [file mmc1.docx]

Procedure U-OptiView DAB IHC v6 (v1.00.0136)

Benchmark ULTRA IHC

Klinikum Augsburg, Institute of Pathology, Stenglin Strasse 2, 86156 Augsburg, Germany

Protocol Name: P-S-100

1 Paraffin [Selected]

2 Oven [Selected]

3 Heat slides to [60°C] and incubate for [4 minutes] (Baking)

4 Deparaffinisation [Selected]

5 Increase slide temperature from [62°C] to medium temperatures (Deparaffinisation)

6 Pre-primary peroxidase blocker [Selected]

7 Primary antibody [Selected]

8 Apply coverslip, add one drop of [S100 (4C4.9)] (Antibody), and incubate for [0 hours 20 min]

9 OptiView HQ Linker [Selected]

10 OptiView HQ Universal Linker [Selected]

11 Apply coverslip, add one drop of OV HQ UNIV LINKR, and incubate for [8 minutes]

12 OptiView HRP Multimer [Selected]

13 HRP Multimer incubation time [Selected]

14 Apply coverslip, add one drop of OV HRP MUL TIMER, and incubate for [8 minutes]

15 Counterstaining [Selected]

16 Apply 1 drop of [HEMATOXYLIN] (Counterstaining), apply LCS, and incubate for [12 minutes]

17 Post-counterstaining [Selected]

18 Apply 1 drop of [BLUING REAGENT] (Post-counterstaining), apply LCS, and incubate for [8 minutes]

Procedure U-OptiView DAB IHC v6 (v1.00.0136)

Benchmark ULTRA IHC

Klinikum Augsburg, Institute of Pathology, Stenglin Strasse 2, 86156 Augsburg, Germany

Protocol Name: P-Synaptophysin

1 Paraffin [Selected]

2 Oven [Selected]

3 Heat slides to [60°C] and incubate for [4 minutes] (Baking)

4 Deparaffinisation [Selected]

5 Increase slide temperature from [62°C] to medium temperatures (Deparaffinisation)

6 Heat pre-treatment [Selected]

7 Ultra CC1 [Selected]

8 Heat slides to [95°C] and incubate for 4 minutes (Cell Conditioner No. 1)

9 CC1 8 min [Selected]

10 CC1 16 min [Selected]

11 CC1 24 min [Selected]

12 Pre-primary peroxidase blocker [Selected]

13 Primary antibody [Selected]

14 Apply coverslip, add one drop of [PREP KIT 137] (Antibody), and incubate for [0 hours 16 min]

15 OptiView HQ Linker [Selected]

16 OptiView HQ Universal Linker [Selected]

17 Apply coverslip, add one drop of OV HQ UNIV LINKR, and incubate for [8 minutes]

18 OptiView HRP Multimer [Selected]

19 HRP Multimer incubation time [Selected]

20 Apply coverslip, add one drop of OV HRP MULTIMER, and incubate for [8 minutes]

21 Counterstaining [Selected]

22 Apply 1 drop of [HEMATOXYLIN] (Counterstaining), apply LCS, and incubate for [12 minutes]

23 Post-counterstaining [Selected]

24 Apply 1 drop of [BLUING REAGENT] (Post-counterstaining), apply LCS, and incubate for [8 minutes]

Procedure U-OptiView DAB IHC v6 (v1.00.0136)

Benchmark ULTRA IHC

Klinikum Augsburg, Institute of Pathology, Stenglin Strasse 2, 86156 Augsburg, Germany

Protocol Name: P-Calretinin

1 Paraffin [Selected]

2 Oven [Selected]

3 Heat slides to [60°C] and incubate for [4 minutes] (Baking)

4 Deparaffinisation [Selected]

5 Increase slide temperature from [62°C] to medium temperatures (Deparaffinisation)

6 Heat pre-treatment [Selected]

7 Ultra CC1 [Selected]

8 Heat slides to [100°C] and incubate for 4 minutes (Cell Conditioner No. 1)

9 CC1 8 min [Selected]

10 CC1 16 min [Selected]

11 CC1 24 min [Selected]

12 CC1 32 min [Selected]

13 Pre-primary peroxidase blocker [Selected]

14 Primary antibody [Selected]

15 Apply coverslip, add one drop of [Calretinin SP65] (Antibody), and incubate for [0 hours 24 min]

16 Counterstaining [Selected]

17 Apply 1 drop of [HEMATOXYLIN] (Counterstaining), apply LCS, and incubate for [12 minutes]

18 Post-counterstaining [Selected]

19 Apply 1 drop of [BLUING REAGENT] (Post-counterstaining), apply LCS, and incubate for [8 minutes]
