# Supplementary material for: Morphological features and molecular mechanisms in peritoneal adhesions from patients with chronic abdominal postoperative pain
Source: eBioMedicine. 2025 May 23;116:105746. doi: 10.1016/j.ebiom.2025.105746 (PMC12153383; doi:10.1016/j.ebiom.2025.105746)
Supplement: Supplementary Information Protocol Pain Pad [file mmc2.docx]

SUPPLEMENTARY INFORMATION

Step-by-step protocol for the mRNA analysis of the studied genes using rt-qPCR:

RNA isolation was performed using the Qiagen micro kit (Cat. No. 74004, Qiagen). From each sample, the adhesion was thawed and taken out of the RNAlater®. 8 mg tissue was excised from the total adhesion. Remaining tissue was restored in RNAlater® at -80 °C. The excised tissue was crushed with a pestle and mortar. This was done with the use of liquid nitrogen to keep the sample cold and prevent RNAse activation. The crushed sample was diluted in 150 microliters of RLT buffer (supplied by kit), which inactivated the RNAses and lyses cells, the solution was put through a 20 gauge needle and syringe. After the solution was homogenised it was treated with proteinase K (cat. No. 19131, Qiagen). Cells were incubated with proteinase K (0.22 mg/ml) for 10 minutes at 55°C . Whereafter, the whole sample was centrifuged for 3 minutes at 10.000xg The supernatant was added to 225 microliter of 100% ethanol and the solution was transferred to a RNeasy minElute spin column in a 2 ml collection tube.

In the column, a series of wash and spin steps were performed. First the complete solution was spun down for 3 min at 10.000xg,. Secondly, 350 µl RW1 buffer (supplied by kit) was added and spun down for 15 seconds at >8000xg, the flowthrough was discarded. After this step, the column was treated with a DNase mixture, containing Dnase and RDD buffer (Qiagen Dnase kit. Cat. No. 79256, Qiagen). Following an incubation period of 15 minutes, 350 µl RW1 buffer was added and the column was spun down for 15 seconds at >8000xg. The flowthrough and collection tube were discarded and the column was put in a new collection tube. 500 µl of RPE buffer (supplied by kit) was added which removes traces of salt. The column was again spun down for 15 seconds at >8000xg and the flow through was discarded. Next, 500 µl of 80% ethanol was added and spun down for 2 minutes at >8000xg. Again, flowthrough and collection tube were discarded. Remaining ethanol was pipetted off the side of the column after which the columns were spun down for 5 minutes at full speed (12.500xg) with an open lid to remove all ethanol.

RNA was eluted in 18 µl RNase free water. 1 µl of the RNA elution was used for a nanodrop (ND-1000 UV-Vis Spectrophotometer, Thermo fisher scientific) to measure the concentration of the RNA. RNA samples were stored at -80°C before use in cDNA synthesis.

cDNA synthesis

Previously isolated RNA was used to create cDNA with the use of the iScript cDNA synthesis kit, according to manufacturer’s instructions. (cat. No. 1708891, BioRAD). The reaction mixture consisted of 4 µl 5x iScript Reaction Mix and 1 µl iScript Reverse Transcriptase, 350 ng RNA template and nuclease-free water in a total volume of 20ul. . Samples that did not yield a total of 350 ng RNA were excluded from further analysis at this time point.

cDNA synthesis protocol was run in a Bio-Rad thermocycler T100 (ID 68567) following the iScript cDNA synthesis kit’s protocol which consisted of a 5 minutes priming step at 25 °C, a 20 minutes reverse transcriptase step at 46 °C and a 1 minute step at 95 °C to inactivate the reverse transcriptase. Samples were stored at -20 °C until further use.

Primer validation

Most of the primers were designed using Primer blast, with the exception of BDNF ^17^ and SRPK2 ^18^, which were based on literature. NGF primers were obtained from the Harvard primer bank. Primers preferably had a melting temperature between 59.0 °C and 61.0 °C, with a product length of 40-150 base pairs (bp). Furthermore, primers had to be specific for the target of interest. Primer sequences can be found in appendix 1.

All primers, except for NGF, were validated with cDNA derived from CaCo2 cells (provided by department of MDL, Daisy Dalloyaux), as these cells expressed the genes of interest. . NGF is mainly expressed in ovarian tissue, heart muscle and in adipose tissue. As some of the adhesion samples were composed majorly of adipose tissue, NGF was validated with the use of a fatty adhesion sample. To create a validation curve for each primer, a series of dilutions was made starting at 1:2.5 or 1:5 dilution and was diluted 2x every two wells. Samples were measured in duplicate and a no template control was taken along, also in duplicate. Primers with a specificity between 90%-110% and a E-value close to 1 were considered to be validated.

RT-qPCR

The cDNA was later used to perform a Real Time quantitative Polymerase Chain Reaction (RT-qPCR). A Biorad 96-well plate (cat. No. HSP9601/ HSP9631) was used to measure the fluorescence of the samples. Seven genes were measured which were: TRPV1, TAC1, TACR1, BDNF, SRPK2 & PDE4D. Three housekeeping genes were also measured which were: RPS11, 18S and HPRT. Plate setups were made according to the sample maximisation strategy. Each plate included 1 gene. On the plate 30 samples were loaded, as well as an internal control to account for run-run variability, and a no template control to confirm potential contamination. Samples were measured in triplicate to compensate for the fact that adhesion tissue is quite heterogenous. Each well contained 2.5 µl cDNA and 10 µl mastermix. The mastermix consists of 6.25 µl SYBRgreen (ref. nr. A25742), which contained all essentials for a qPCR, 0.5 µl of both the forward and reversed primer and 2.75 µl milliQ to create a total volume of 10 µl. RT qPCR was run on a Biorad CFX connect (ID 63181). The qPCR was run consisting of 7 minutes denaturation period at 95°C, followed by 40 amplification cycles consisting of 15 seconds at 95 °C and a 1 minute annealing period at 60 °C. Subsequently, a melt curve was made.
