# Supplementary material for: Morphological features and molecular mechanisms in peritoneal adhesions from patients with chronic abdominal postoperative pain
Source: eBioMedicine. 2025 May 23;116:105746. doi: 10.1016/j.ebiom.2025.105746 (PMC12153383; doi:10.1016/j.ebiom.2025.105746)

Supplementary Material

Gastroenterology

**Supplementary Figure 1:** (a) Adhesion biopsy approach with atraumatic grasper and cut with non-coagulating scissors (b) Areas for recording location of the pain and adhesions within the abdomen

(a)
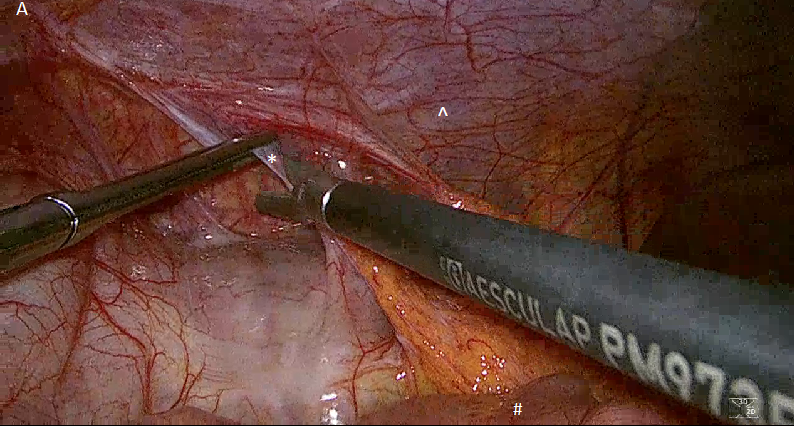


* = Adhesion # = Bowel with mesentery attached ^ = Abdominal wall (right sided)

(b)


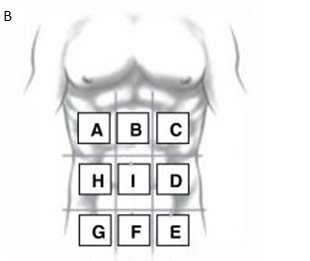


**Supplementary Figure 2:**  Examples of scanned samples of adhesion biopsies. (a) A sample coloured using H&E staining; with randomly placed stereotactic point counting arrows, surrounded by annotation to define the boundaries of the adhesion. (digitised at 40x magnification using an Aperio T2 scanner (Leica Microsystems)) (b+c) Example of colour detection method for S100 IHC staining, including the grey colour thresholds (19-87) (digitised at 40x magnification using an Aperio T2 scanner (Leica Microsystems)) (b) and view mode (c).

(a)


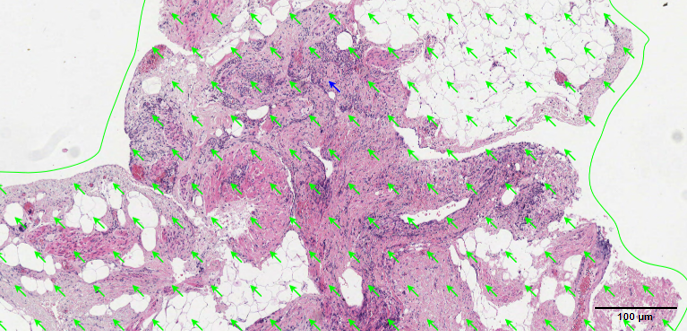


(b) (c)


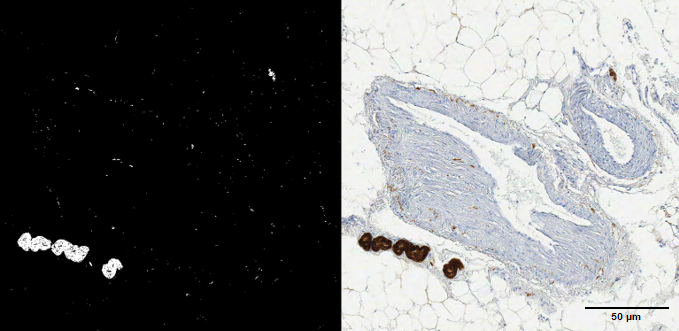


**Supplementary Table 1:** Primer sequences and validation properties

| Primer | Forward primer | Reverse primer | Amplicon length (bp) | E-value | R^2 value | Linear dynamic range (cq) |
| --- | --- | --- | --- | --- | --- | --- |
| RPS11 | 5’ AGAGGACCATTGTCATCCGC | 5’ AGACATGTTCTTGTGGCGCT | 83 | 96,6% | 0.994 | 15.27 – 20.72 |
| HPRT | 5’ GGATTTGAAATTCCAGACAAGTTT | 5’ GCGATGTCAATAGGACTCCAG | 171 | 91.8% | 0.993 | 25.38 – 29.42 |
| 18S | 5’ AAACGGCTACCACATCCAAG | 5’ CGCTCCCAAGATCCAACTAC | 250 | 98.5% | 0.990 | 24.48 – 28.55 |
| TRPV1 | 5’ TCTCACCTACATCCTCCTGCTCAA | 5’ TTGCTCTCCTGTGCGATCTTGT | 78 | 90.5% | 0.983 | 28.81 – 34.47 |
| TAC1 | 5’ CAGCGACCAGATCAAGGAGG | 5’ CGGGCGATTCTCTGCAGA | 110 | 107.3% | 0.992 | 26.01 – 30.79 |
| TACR1 | 5’ ATGATCGAATGGCCAGAGCAT | 5’ GGAGGAAGTAGATCAGCACAGT | 103 | 97.7% | 0.984 | 28.35 – 33.63 |
| BDNF | 5’ CATCCGAGGACAAGGTGGCTTGG | 5’ GTCCTCATCCAACAGCTCTTCTATC | 148 | 97.1% | 0.979 | 26.44 – 32.07 |
| NGF | 5’ GGCAGACCCGCAACATTACT | 5’ CACCACCGACCTCGAAGTC | 135 | 96.7% | 0.971 | 30.29 – 36.11 |

RPS11, HPRT and 18S = housekeeping genes

TRPV1 = Transient receptor potential vanilloid 1 TAC1=Tachykinin precursor 1 TACR1= Tachykinin receptor 1

BDNF = Brain-derived neurotrophic factor NGF = Nerve Growth Factor

**Supplementary Figure 3** - Representative image of the histology of an abdominal adhesion from one patient (a) an adhesion slide, vascularised to a high degree in different magnifications (digitised at 40x magnification using an Aperio T2 scanner (Leica Microsystems)) (b) with the presence of large blood vessels in different digital magnifications (digitised at 40x magnification using an Aperio T2 scanner (Leica Microsystems)) (c) example of foreign material found in the adhesion sample in different
magnifications.


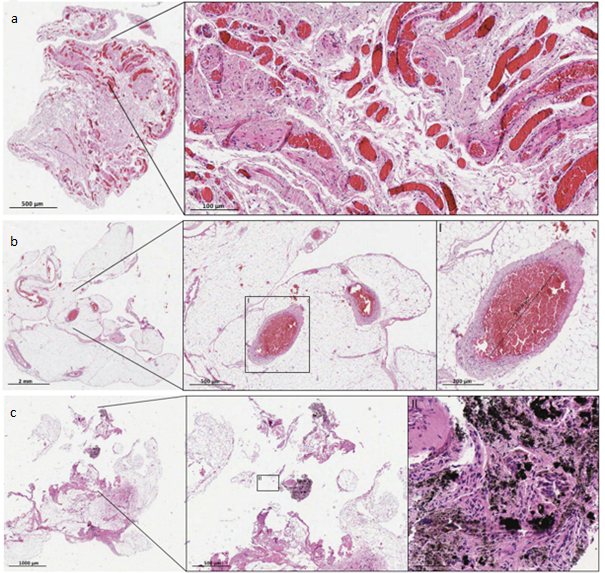


**Supplementary Table 2:** Subanalysis of composition of adhesion biopsies compared between adhesion biopsies taken from the target area of patients with pain, biopsies taken from a non-target area of patients with pain, biopsies of patients with diffuse pain and controls, using H&E staining and assessed by stereotypical point counting. (A one-way analysis of variance (ANOVA))

|  | Target (N = 17)  *Median (range)* | Non-target (N = 8)  *Median (range)* | Diffuse (N = 3)  *Median (range)* | Controls (N = 27)  *Median (range)* | P value |
| --- | --- | --- | --- | --- | --- |
| Blood vessels | 3.8% (0%-12.5%) | 3.0% (0.9%-8.4%) | 1.4% (0.4%-2.5%) | 4.3% (0%-30.7%) | 0.412 |
| Muscle fibres | 0.4% (0%-29.8%) | 0.2% (0%-6.6%) | 6.3% (0%-12.5%) | 1.9% (0%-47.6%) | 0.322 |
| Connective tissue | 47.3% (6.7%-95.3%) | 59.1% (17.6%-96.2%) | 49.4% (37.3%-61.4%) | 49.8% (9.9%-94.6%) | 0.869 |
| Adipose tissue | 50.8% (0%-84.6%) | 33.0% (0%-80.0%) | 41.8% (25.0%-58.5%) | 33.1% (0%-86.3%) | 0.740 |
| Inflammation | 0.4% (0%-1.6%) | 0.4% (0%-5.8%) | 0% (0%-1.7%) | 0.4% (0%-8.6%) | 0.736 |
| Other | 0% (0%-3.2%) | 0% (0%-0.5%) | 0.5% (0%-0.7%) | 1.5% (0%-11.1%) | 0.018 |

**Supplementary Table 3:** Subanalysis of proportion of marker positivity indicating nerve fibres compared between adhesion biopsies taken from the target area of patients with pain (n=17), biopsies taken from a non-target area of patients with pain (n=8), biopsies of patients with diffuse pain (n=3) and controls (n=27). (A one-way analysis of variance (ANOVA))

|  | Target (n=17) *Median (range)* | Non-target (n=8) *Median (range)* | Diffuse (n=3) *Median (range)* | Controls (n=27) *Median (range)* | *P* value |
| --- | --- | --- | --- | --- | --- |
| S100, ppm | 608.5 (0-1835.4) | 276.4 (0-3223.2) | 701.9 (0-732.2) | 150.7 (0-1683.8) | 0.327 |
| Calretinin, ppm | 733.7 (0-2996.5) | 258.9 (0-661.4) | 112.0 (0-382.2) | 224.7 (0-3194.8) | 0.057 |
| Synaptophysin, ppm | 743.2 (0-6492.5) | 750.9 (208.6-2263.8) | 84.5 (0-337.9) | 560.1 (0-2040.3) | 0.148 |

**Supplementary table 4:** Intrapatient (n=3) subanalysis of composition of adhesion biopsies compared between adhesion biopsies taken from the target area and non-target of the same patient with pain, using H&E staining and assessed by stereotypical point counting (Independent t-test)

|  | **Target area (n= 3)** *Median% (range)* | **Non-target area (n=3)** *Median% (range)* | ***P* value** |
| --- | --- | --- | --- |
| ***Blood Vessel*** | 2.22% (1.46-4.03%) | 1.24% (0.88-2.28%) | 0.273 |
| ***Muscle Fibres*** | 1.83% (0.00-5.85%) | 0.00% (0.00-6.61%) | 0.904 |
| ***Connective Tissue*** | 33.17% (30.22-83.15%) | 23.29% (22.91-34.16%) | 0.277 |
| ***Adipose Tissue*** | 58.05% (4.76-67.11%) | 69.16% (62.73-73.52%) | 0.271 |
| ***Inflammation*** | 0.98% (0.00-1.83%) | 0.46% (0.44-0.62%) | 0.465 |
| ***Other*** | 0.49% (0.44-4.03%) | 0.46% (0.00-1.24%) | 0.431 |

**Supplementary table 5:** Baseline characteristics of patients with pain (n=29) compared to controls (n=23) from whom the RNA expression levels of adhesion biopsies could be analysed. (Continuous variables are analysed using Mann-Whitney U Test, categorical variables using Chi-Square test)

|  | **Patients with pain n=29** | **Controls n=23** | **Total cohort n=51** | ***P* value** |
| --- | --- | --- | --- | --- |
| **Sex**, n (%)  Females  Males | 23 (79%)  6 (21%) | 12 (52%)  11 (48%) | 35 (67%)  17 (33%) | 0.022 |
| **History of small bowel obstruction**, n (%) | 10 (36%) | 4 (18%) | 14 (28%) | 0.215 |
| **Malignancy**, n (%) | 5 (17%) | 10 (43%) | 15 (29%) | 0.063 |
| **Peritoneal Adhesion Index (PAI)**, median (range) | 7.5 (2-30) | 6.0 (1-30) | 6.0 (1-30) | 0.464 |

**Supplementary figure 4:** Detection rates of target genes using rt-PCR of subanalysis comparing biopsies of patients with pain taken from a target area (n=26), biopsies from patients with pain but taken from a non-target area (n=10), and biopsies from patients with diffuse pain (n=4) to the biopsies of controls (n=30). (a) Subanalysis of detection rates of transient receptor potential vanilloid 1 (TRPV1) comparing biopsies of patients with pain taken from a target area (n=26), biopsies from patients with pain but taken from a non-target area (n=10), and biopsies from patients with diffuse pain (n=4) to the biopsies of controls (n=30) (b) Subanalysis of detection rates of tachykinin precursor 1 (*TAC1*) encoding for substance P (SP) comparing biopsies of patients with pain taken from a target area (n=26), biopsies from patients with pain but taken from a non-target area (n=10), and biopsies from patients with diffuse pain (n=4) to the biopsies of controls (n=30) (c) Subanalysis of detection rates of tachykinin receptor 1 (*TACR1*) encoding for neurokinin 1 receptor (NK1R) comparing biopsies of patients with pain taken from a target area (n=26), biopsies from patients with pain but taken from a non-target area (n=10), and biopsies from patients with diffuse pain (n=4) to the biopsies of controls (n=30) (d) Subanalysis of detection rates of brain-derived neurotrophic factor (*BDNF*) comparing biopsies of patients with pain taken from a target area (n=26), biopsies from patients with pain but taken from a non-target area (n=10), and biopsies from patients with diffuse pain (n=4) to the biopsies of controls (n=30) (e) Subanalysis of detection rates of nerve growth factor (*NGF*) comparing biopsies of patients with pain taken from a target area (n=26), biopsies from patients with pain but taken from a non-target area (n=10), and biopsies from patients with diffuse pain (n=4) to the biopsies of controls (n=30)


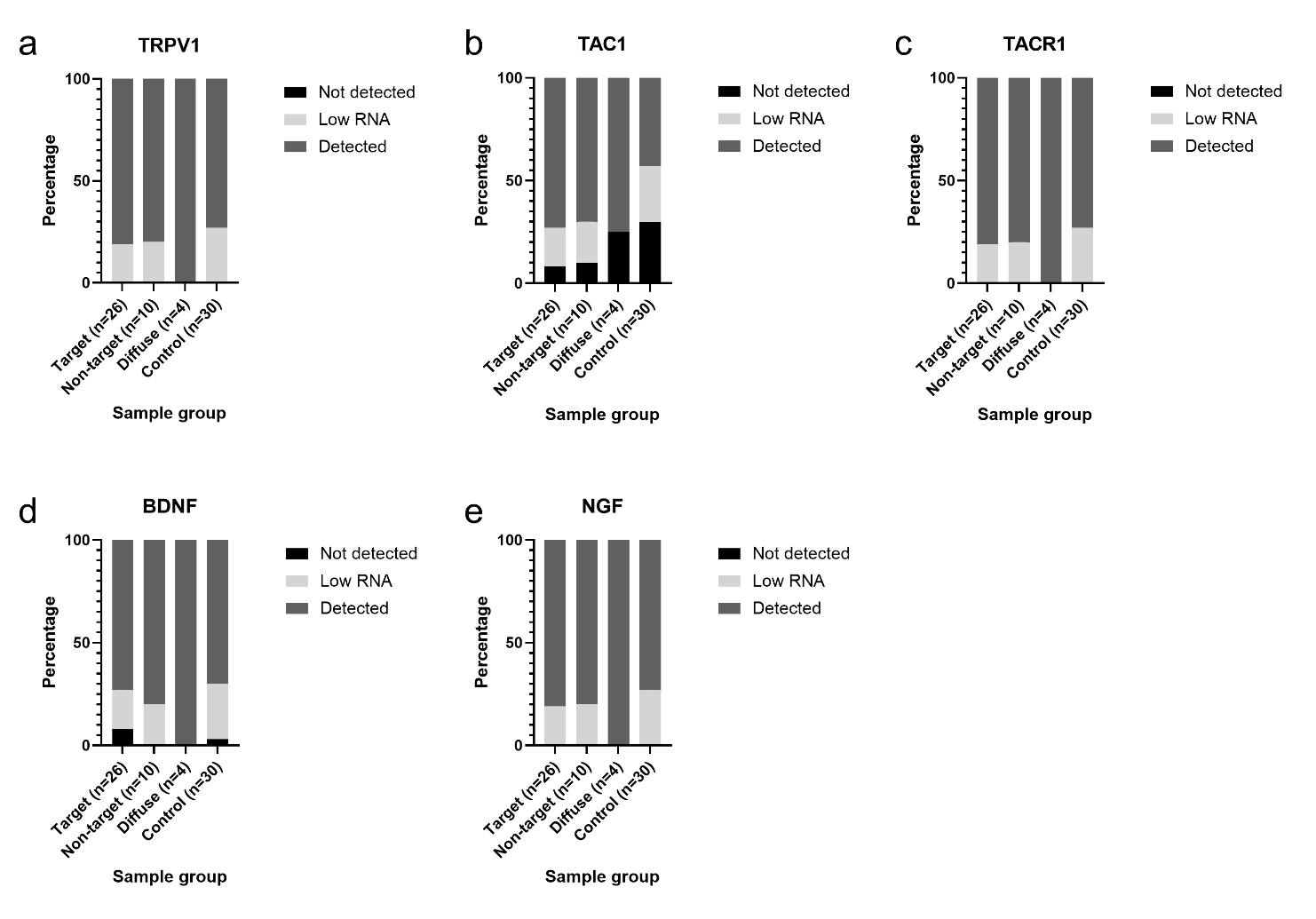


**Supplementary figure 5:** Expression levels of target genes using rt-PCR of subanalysis comparing biopsies of patients with pain taken from a target area (n=21), biopsies from patients with pain but taken from a non-target area (n=8), biopsies from patients with diffuse pain (n=4) were compared to biopsies of controls (n=22) (a) Subanalysis of expression levels of transient receptor potential vanilloid 1 (TRPV1) comparing biopsies of patients with pain taken from a target area (n=21, p=0,655), biopsies from patients with pain but taken from a non-target area (n=8, p=0,599), biopsies from patients with diffuse pain (n=4, p=0.998) were compared to biopsies of controls (n=22) (a one-way analysis of variance (ANOVA)) (b) Subanalysis of expression levels of tachykinin precursor 1 (*TAC1*) encoding for substance P (SP) comparing biopsies of patients with pain taken from a target area (n=21, p>0.999), biopsies from patients with pain but taken from a non-target area (n=8p=0.813), biopsies from patients with diffuse pain (n=4,p=0.913) were compared to biopsies of controls (n=22) (A one-way analysis of variance (ANOVA)) (c) Subanalysis of expression levels of tachykinin receptor 1 (*TACR1*) encoding for neurokinin 1 receptor (NK1R) comparing biopsies of patients with pain taken from a target area (n=21, p=0.988), biopsies from patients with pain but taken from a non-target area (n=8, p=0.120), biopsies from patients with diffuse pain (n=4, p=0.089) were compared to biopsies of controls (n=22) (A one-way analysis of variance (ANOVA)) (d) Subanalysis of expression levels of brain-derived neurotrophic factor (*BDNF*) comparing biopsies of patients with pain taken from a target area (n=21, p=0.999), biopsies from patients with pain but taken from a non-target area (n=8,p0.952), biopsies from patients with diffuse pain (n=4p=0.997) were compared to biopsies of controls (n=22) (A one-way analysis of variance (ANOVA)) (e) Subanalysis of expression levels of nerve growth factor (*NGF*) comparing biopsies of patients with pain taken from a target area (n=21,p=0.031), biopsies from patients with pain but taken from a non-target area (n=8, p=0.409), biopsies from patients with diffuse pain (n=4, p=0.945) were compared to biopsies of controls (n=22) (A one-way analysis of variance (ANOVA))


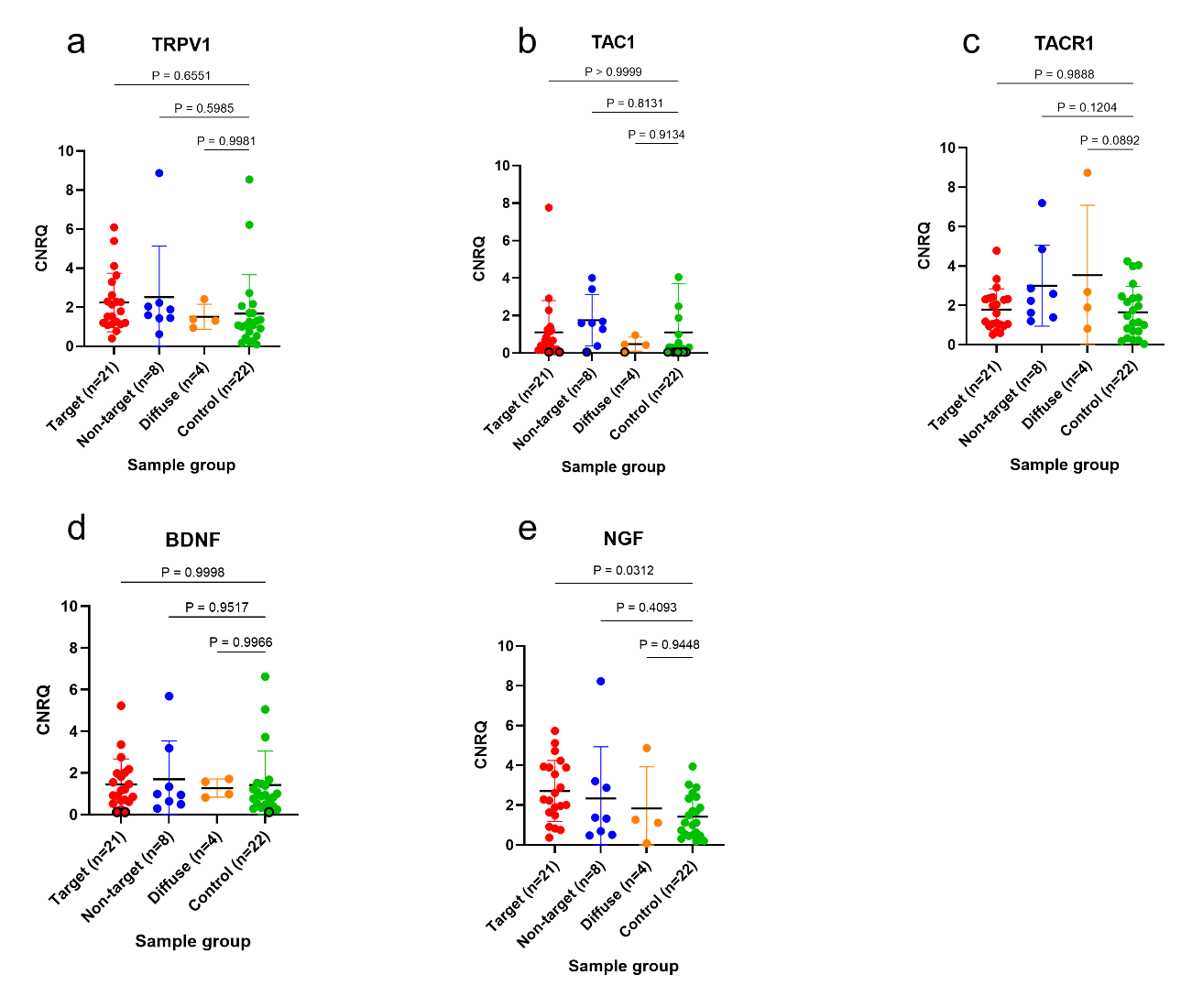


**Supplementary figure 6:** Intrapatient subanalysis of detection rates of biopsies from patients with pain in whom both a biopsy from a target area and a non-target area was taken (n=6), using RT-qPCR. (a) Intrapatient (n=6) subanalysis of detection rates of transient receptor potential vanilloid 1 (TRPV1) (b) Intrapatient (n=6) subanalysis of detection rates of tachykinin precursor 1 (*TAC1*) encoding for substance P (SP) (c) Intrapatient (n=6) subanalysis of detection rates of tachykinin receptor 1 (*TACR1*) encoding for neurokinin 1 receptor (NK1R) (d) Intrapatient (n=6) subanalysis of detection rates of brain-derived neurotrophic factor (*BDNF*) (e) Intrapatient (n=6) subanalysis of detection rates of nerve growth factor (*NGF*) **
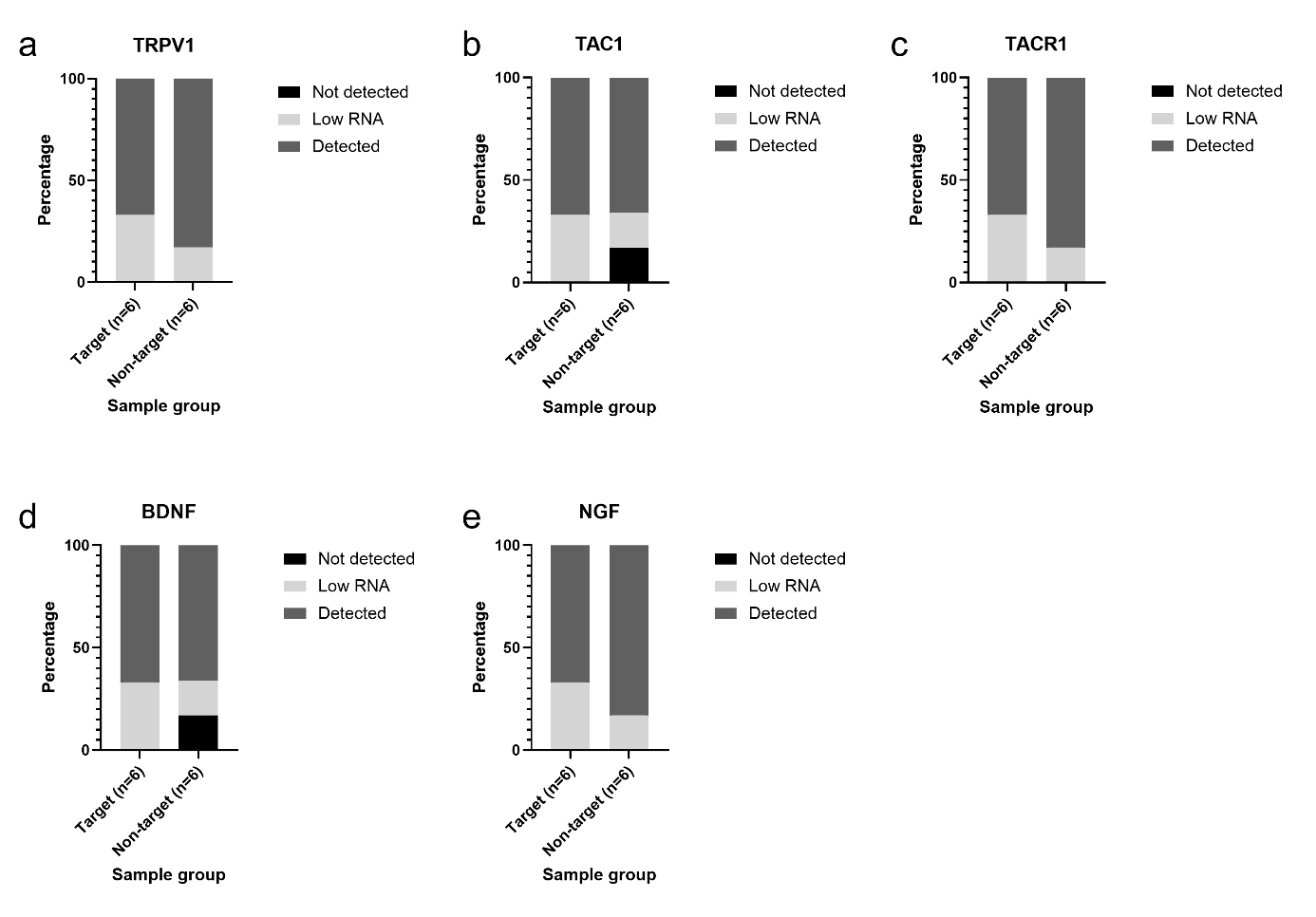
**

**Supplementary figure 7:** Intrapatient subanalysis of expression levels of biopsies from patients with pain in whom both a biopsy from a target area and a non-target area was taken (n=6), using RT-qPCR. (a) Intrapatient (n=6) subanalysis of expression levels of transient receptor potential vanilloid 1 (TRPV1), compared between a biopsy from a target area and a non target area (p=0.672, two-tailed t-test) (b) Intrapatient (n=6) subanalysis of expression levels of tachykinin precursor 1 (*TAC1*) encoding for substance P (SP), compared between a biopsy from a target area and a non target area (p=0.183, two-tailed t-test) (c) Intrapatient (n=6) subanalysis of expression levels of tachykinin receptor 1 (*TACR1*) encoding for neurokinin 1 receptor (NK1R), compared between a biopsy from a target area and a non target area (p=0.415, two-tailed t-test) (d) Intrapatient (n=6) subanalysis of expression levels of brain-derived neurotrophic factor (*BDNF*), compared between a biopsy from a target area and a non target area (p=0.906, two-tailed t-test) (e) Intrapatient (n=6) subanalysis of expression levels of nerve growth factor (*NGF*), compared between a biopsy from a target area and a non target area (p=0.040, two-tailed t-test)


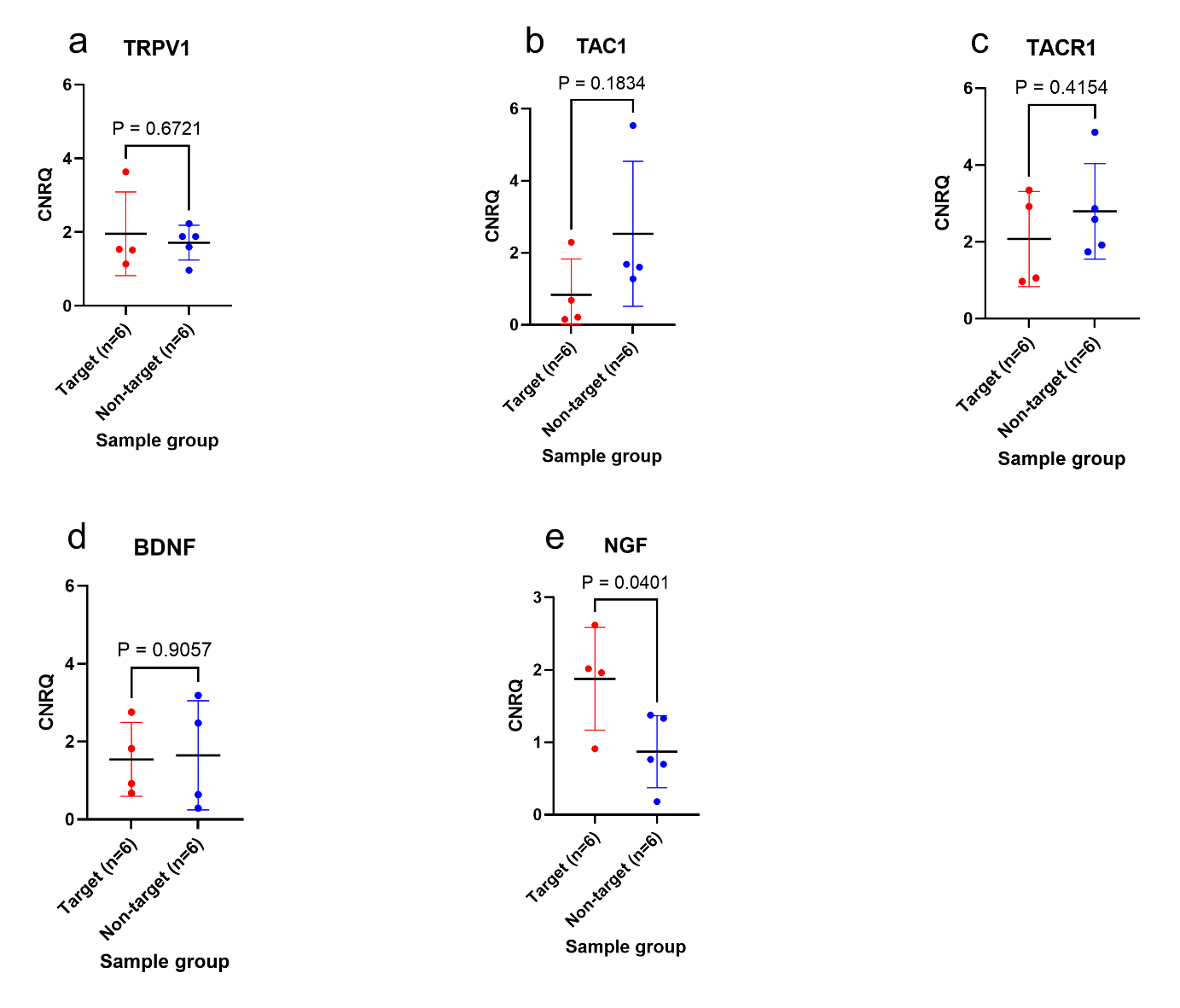

Supplement: Supplementary Figures and Tables [file mmc3.docx]
